# Structural Characterization and Molecular Docking Studies of Fresh Coconut Meat Polysaccharides

**DOI:** 10.3390/ijms262010222

**Published:** 2025-10-21

**Authors:** Jiayuan Huang, Mingyang Ma, Miaomiao Qin, Xinyun Li, Yongshen Ren

**Affiliations:** 1 Institute of Drug Discovery Technology, Ningbo University, Ningbo 315211, China; 17733166968@163.com (J.H.);; 2 School of Pharmacy, Hainan University, Haikou 570228, China

**Keywords:** fresh coconut meat, polysaccharides, structural characterization, molecular docking

## Abstract

Fresh coconut meat polysaccharides (FCMPs) are high-value natural active polysaccharides with both medicinal and edible uses, but their structural characteristics and potential biological activities have not been well studied. In this work, FFCMP was separated and purified by sequential application of water extraction and alcohol precipitation methods, the Sevag method, DEAE-52 cellulose column chromatography, and Sephadex G-100 gel column chromatography, yielding four components (FCMP 1-FCMP 4). High-performance liquid chromatography (HPLC) was used to determine their molecular weights as 343,016.9, 2279.4, 1363.2, and 2228.9 Da, respectively. Structural characterization and monosaccharide analysis revealed that the FCMP series primarily consists of mannose, glucose, galactose, arabinose, and rhamnose. Methylation experiments and nuclear magnetic resonance (NMR) indicated that FCMP 1 exhibits a complex topological structure with a β-1→4 main chain, β-1→6 branches, and an α-L-rhamnose terminal; FCMP 2 is a heteropolysaccharide with a β-(1→3)-mannan main chain containing β-(1→6)-galactose branches; the main chain of FCMP 3 consists of β-D-mannose and β-D -galactose, with side chains containing α-L-rhamnose and terminal α-L-arabinose and β-D-mannose; and FCMP 4 has a main chain primarily composed of glucose and mannose linked via 1→4 bonds, with some C6 positions exhibiting 1→6 branch structures. Molecular docking predictions suggest that the FCMP series of polysaccharides possess immunomodulatory, anti-inflammatory, and edema-treating properties, providing a theoretical basis for their application in pharmacology and food science research.

## 1. Introduction

Structure–Activity Relationship and Potential Bioactivities of Fresh Coconut Meat Polysaccharides (FCMPs): As an important class of biomacromolecules, FCMPs hold significant application value in the food, pharmaceutical, and health product industries. In recent years, polysaccharides isolated from natural resources—particularly plant-derived polysaccharides—have attracted considerable attention due to their structural diversity and multifunctionality, demonstrating remarkable biological activities such as immunomodulation, anti-inflammatory effects, and antioxidant properties [1].

Recent studies have revealed that Polygonatum sibiricum water extracts exhibit a broad spectrum of bioactivities, including hepatoprotection, antioxidant effects, and anti-inflammatory activity [2,3,4,5,6]. Pharmacological investigations have shown that polysaccharides derived from Cordyceps sinensis possess immunomodulatory, antitumor, hypoglycemic, and antifibrotic effects [7]. Furthermore, modern pharmacological research has demonstrated that Sargassum pallidum polysaccharides exhibit a wide range of health-promoting properties, such as antioxidant, anticancer, hypolipidemic, and immunomodulatory activities [8,9,10,11]. However, due to the intricate molecular weight hierarchy and unresolved mechanisms of action—particularly regarding how polysaccharides and oligosaccharides with polydisperse molecular weights exert their bioactivities—the structure–activity relationship remains to be further elucidated [12].

FCMPs, as bioactive components derived from coconut processing by-products, a tropical specialty resource, have been reported to possess various biological activities, including immunomodulation, anti-inflammation, and antioxidant effects [13,14,15]. Nevertheless, the fine structural characteristics of FCMP extracts and their relationship with bioactivities have not been systematically investigated. The polysaccharide components of FCMPs are complex, with a broad molecular weight distribution, and different fractions may exhibit distinct biological activities and mechanisms of action [16].

In this study, we employed modern separation and purification techniques to fractionate FCMPs and applied multiple structural characterization methods (e.g., infrared spectroscopy, nuclear magnetic resonance, and methylation analysis) to elucidate their fine structures. Additionally, molecular docking simulations were performed to predict the interactions between FCMP fractions and immune-related targets (e.g., TLR4/MD-2 complex, DC-SIGN receptor, AQP1), aiming to explore their potential bioactivities. The findings of this study will provide new insights into the structure–function relationship of FCMPs and lay a foundation for their application in immunomodulation, anti-inflammatory therapy, and antioxidant interventions.

## 2. Results and Analysis

### 2.1. Molecular Weight Determination Results

Based on the results obtained using the dextran standard, the standard logarithmic curve is plotted as y = −3.0907x + 49.748, R^2^ = 0.9978.

**Figure 1:**
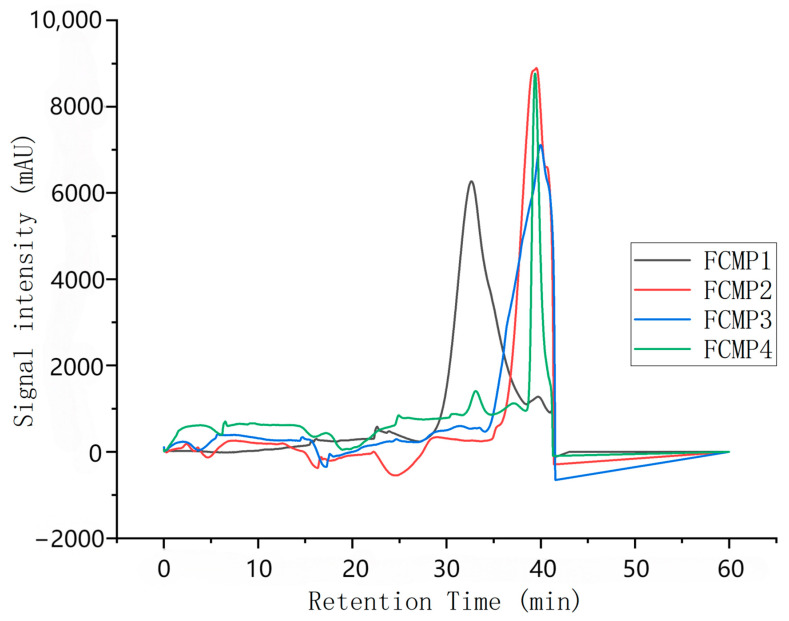
The molecular weight map of purified polysaccharides at different levels.

As shown in Figure 1, the retention times of FCMP 1–4 are 32.64 min, 39.37 min, 40.06 min, and 39.40 min, respectively. Substituting these values into the standard curve, the calculated molecular weights are 343,016.9, 2279.4, 1363.2, and 2228.9, respectively.

These molecular weight data indicate that FCMP 1 has a significantly higher molecular weight than FCMP 2, FCMP 3, and FCMP 4, which may suggest that FCMP 1 has a more complex structure or contains more long-chain polysaccharide molecules. The molecular weights of FCMP 2, FCMP 3, and FCMP 4 are relatively close, which may indicate that they share structural similarities or originate from similar polysaccharide components.

### 2.2. Infrared Spectroscopy Results

**Figure 2:**
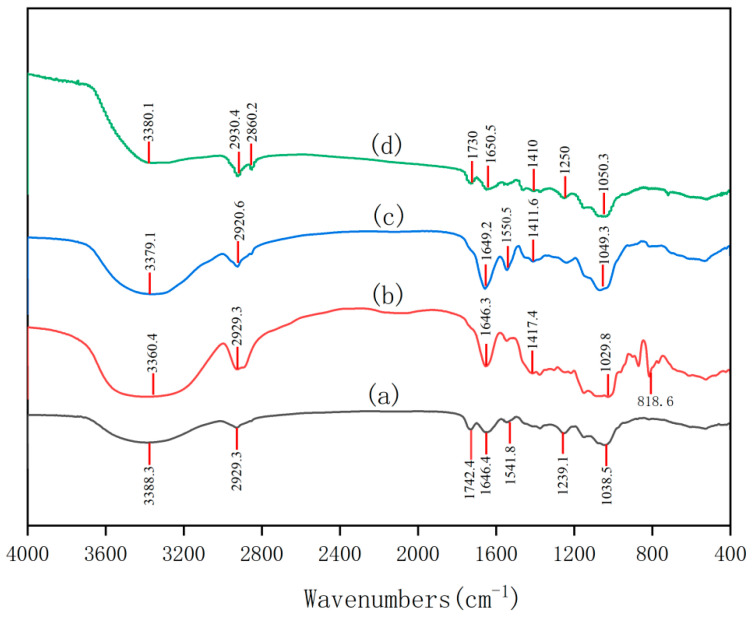
The infrared spectra of polysaccharides at all levels. (a) FCMP 1. (b) FCMP 2. (c) FCMP 3. (d) FCMP 4.

Infrared spectroscopy analysis employs the KBr pellet method, and each absorption peak in the functional group region of the infrared spectrum corresponds to a specific functional group. As shown in Figure 2, the FT-IR spectra of FCMP 1–4 samples all exhibit typical characteristic absorption peaks of polysaccharides. The characteristic peak of polysaccharides at 3300–3400 cm^−1^ corresponds to the stretching vibration of O-H, indicating the presence of abundant hydroxyl (-OH) groups in the samples, which is a core characteristic of polysaccharide structure. The absorption peak at 2920–2930 cm^−1^ corresponds to the stretching vibration of C-H bonds, further confirming the presence of C-H bonds in the polysaccharide sugar rings [17].

The characteristic absorption peaks at 1710–1750 cm^−1^ and 1649–1690 cm^−1^ can be attributed to C=O stretching vibrations, indicating that the sample may contain ester groups (-COOR) or carboxyl groups (-COOH), suggesting that these polysaccharides possess acidic properties [18]. The absorption peak near 1400–1420 cm^−1^ may be related to N-H bending vibrations, suggesting the presence of acetylamino groups (e.g., N-acetylglucosamine) in the polysaccharides, commonly found in polysaccharides such as chitin [19].

The absorption peak at 1100–1280 cm^−1^ corresponds to C-O stretching vibrations, indicating the presence of glycosidic bonds (C-O-C) or hydroxyl groups (C-OH) in the polysaccharides. The characteristic peak at 1038.5 cm^−1^ further supports C-O vibrations, consistent with the C-O-C bonds in the pyranose monosaccharide ring. The absorption peak at 818.6 cm^−1^ can be attributed to C-H bending vibrations, consistent with the typical configuration of D-pyranose glucose. These characteristic peaks collectively confirm the presence of pyranose rings in FCMP 1–4 samples and indicate their structural characteristics as acidic polysaccharides [20].

### 2.3. Results of Monosaccharide Composition Analysis

**Figure 3:**
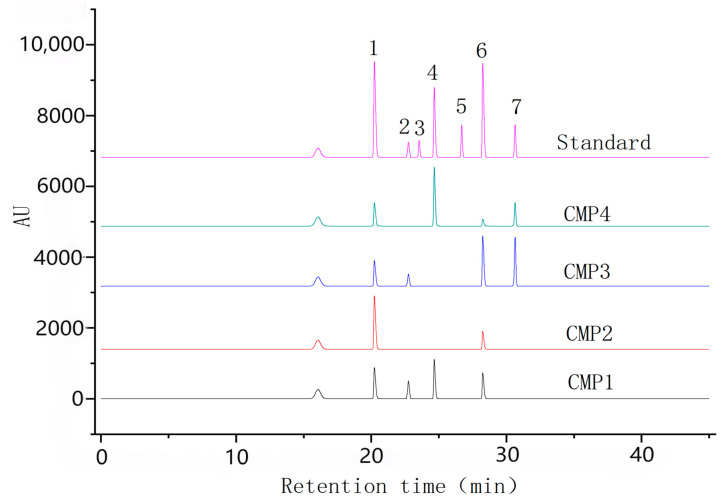
The monosaccharide composition diagram. Note: Standards 1–7 are mannose, rhamnose, galacturonic acid, glucose, sorbitol, galactose, and arabinose, respectively.

In Figure 3, the monosaccharide composition diagram shows that standards 1–7 are, in order, mannose, rhamnose, galacturonic acid, glucose, sorbitol, galactose, and arabinose. From this diagram, it can be seen that FCMP 1 consists of mannose, rhamnose, glucose, and galactose; FCMP 2 consists of mannose and galactose; FCMP 3 consists of mannose, rhamnose, galactose, and arabinose; and FCMP 4 consists of mannose, glucose, galactose, and arabinose.

### 2.4. Methylation Experiment Results

#### 2.4.1. Methylation Results of FCMP 1

The methylation results of FCMP 1 shown in Table 1 indicate that the monosaccharide composition consists of D-mannose (approximately 40%), D-glucose (approximately 30%), D-galactose (approximately 20%), and L-rhamnose (approximately 10%). The main chain is composed of alternating β-1→4-linked mannose and glucose, branches are formed by galactose linked via β-1→6 bonds, and the terminal end is modified by α-L-rhamnose via 1→2/1→3 bonds.

#### 2.4.2. Methylation Results of FCMP 2

As shown in Table 2, the FCMP 2 methylation results indicate that the molar ratio of the sugar residues in FCMP 2 is main chain mannose/branch galactose/terminal mannose = 2:1:1, with a molecular weight of 2279.40 Da. Combining the molecular weights of the methylated monosaccharides (both mannose and galactose are 273.25 Da after methylation) and the dehydration weight of the glycosidic bonds (18.02 Da per bond), it can be inferred that this polysaccharide is composed of eight monosaccharide units, with the main chain containing five β-(1→3)-Manp units (5 × 273.25 = 1366.25 Da), the branches containing two β-(1→6)-Galp units (2 × 273.25 = 546.50 Da), the terminal containing one Manp unit (273.25 Da), and the glycosidic bonds undergoing seven dehydration steps (7 × 18.02 = 126.14 Da), with total molecular weight of 1366.25 + 546.50 + 273.25 − 126.14 ≈ 2060 Da. The actual molecular weight is slightly higher, possibly due to incomplete coverage of some methylation sites or the presence of trace impurities, but the overall structure is consistent with the experimental data [21].

#### 2.4.3. Methylation Results of FCMP 3

As shown in Table 3, the total molar ratio of FCMP 3 sugar residues is D-mannose/D-galactose/L-rhamnose/L-arabinose = 3.16:1.63:4.8:1.60. The main chain is primarily composed of D-mannose and D-galactose, the linkage patterns include 1→3 and 1→6; and the side chains include 1→4 linkages of L-rhamnose and terminal structures of L-arabinose [22].

#### 2.4.4. Methylation Results of FCMP 4

As shown in Table 4 FCMP 4 methylation results, glucose (Glc) forms the main chain through 1→4 glycosidic bonds (retention time 6.899 min, characteristic peak *m*/*z* 126.06, and molar ratio 25.05%), with some containing 1→4,6 branches (retention time 14.069 min, *m*/*z* 199, and molar ratio 8.73%), and terminal Glc (retention time 27.464 min and *m*/*z* 218) indicates the terminal end of the linear chain. Mannose (Man) is primarily present as 1→6 linkages (retention time 6.925 min, *m*/*z* 175.13, and molar ratio 15.34%) and 1→3,6 branches (retention time 17.788 min, *m*/*z* 207, and molar ratio 7.98%). Galactose (Gal) is present in 1→3 linkages (retention time 11.769 min, *m*/*z* 156.1, and molar ratio 10.31%) and 1→2 linkages (retention time 19.038 min, *m*/*z* 154.08, and molar ratio 6.15%). Arabinose (Ara) is primarily present in a 1→5 linkage (retention time 12.947 min, *m*/*z* 149.05, and molar ratio 9.82%) and a 1→3,5 branch (retention time 20.006 min, *m*/*z* 227, and molar ratio 5.21%) [23].

### 2.5. Nuclear Magnetic Resonance Analysis Results

#### 2.5.1. FCMP 1 Magnetic Resonance Analysis Results

Figure S1a shows the ^13^C NMR characteristics: C1 (δ100.17) combined with the H1 coupling constant (J = 8.7 Hz) in ^1^H NMR confirms that the main chain is a β-1→4-linked D-mannose/D-glucose. C4 (δ68.38) and the H1 long-range coupling in HMBC verify the 1→4 bond, C6 (δ61.21) indicates an unsubstituted primary alcohol group, and the branch point C6 (δ68.38) is associated with a β-1→6-linked D-galactose. The terminal α-L-rhamnose is confirmed by the methyl signal (δ1.09/1.66) and the hetero proton (δ4.94, single peak). HMBC shows that it is linked to the main chain via 1→2/1→3 connections [24,25].

^1^H NMR data in Figure S1b: δ4.94 (s, 1H) corresponds to α-L-rhamnose H1 (J ~ 1–2 Hz); δ3.90 (d, J = 8.7 Hz, 2H) is assigned to β-D-glucose/mannose H1; δ3.84 (d, J = 17.7 Hz, 5H) corresponds to the C6-H6a/H6b trans coupling; and δ3.73 (d, J = 9.8 Hz, 4H) corresponds to the β-1→4-linked H4/H5 trans coupling [26,27].

**Figure 4:**
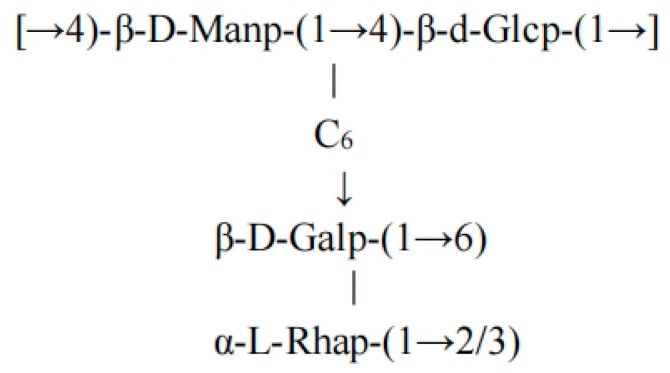
Diagram of the FCMP1 backbone structure.

Based on the methylation analysis and NMR data, FCMP 1 consists of ~40% D-mannose, ~30% D-glucose, ~20% D-galactose, and ~10% L-rhamnose, with the main chain consisting of alternately linked β-1→4 mannose/glucose units, β-1→6 galactose branches, and terminal α-L-rhamnose modified via 1→2/1→3 linkages. The highly branched structure (molecular weight > 300 kDa) exhibits one branch point every 5–6 main chain units [28,29]. In summary, the polysaccharide possesses a complex topology characterized by a β-1→4 glycosidic backbone, β-1→6 glycosidic side chains, and α-L-rhamnose terminal units. The structure of the backbone is depicted in Figure 4.

#### 2.5.2. FCMP 2 Nuclear Magnetic Resonance Analysis Results

**Figure 5:**
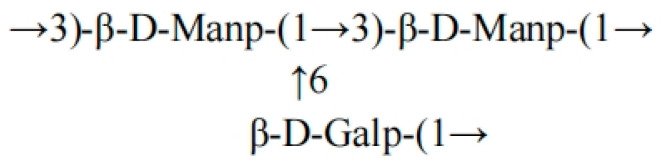
Structural formula of the FCMP 2 backbone.

Figure S2 shows the NMR (^1^H/^13^C/COSY/HSQC) spectrum: the main chain is connected by β-D-Manp (1→3), with C1 (δ99.91) strongly correlated with H1 (δ5.11, dd, J = 16.8 Hz) and the de-shielding effect of C3 (δ74.68) confirming the β-configuration; the branch is connected to the main chain Man by β-D-Galp (1→6), and C1 (δ97.32, H1 δ4.91, J≈7.5 Hz) and Man C6 (δ60.44, H6 δ3.48) chemical shifts support this structure. In the HSQC spectrum, the carbon–hydrogen correlations of Man C2 (δ76.62, H2 δ4.05), C4 (δ72.74, H4 δ3.73), and Gal C6 (δ65.62, H6 δ3.55) in the HSQC spectrum are consistent with the methylation data (*m*/*z* 199.10/232.09), confirming the Man-(1→3)-Man main chain and Gal-(1→6)-Man branch [30,31]. The terminal Man signal (C1 δ93.44, H1 δ4.70) and trace Gal signal (C1 δ98.62) suggest chain termination or incomplete linkage. In summary, FCMP 2 is a heteropolysaccharide with a β-(1→3)-mannan main chain containing β-(1→6)-galactose branches [32,33]. The structural formula of the FCMP 2 backbone is shown in Figure 5.

#### 2.5.3. FCMP 3 Nuclear Magnetic Resonance Analysis Results

Through comprehensive methylation analysis and systematic interpretation of the FCMP 3 nuclear magnetic resonance (NMR) spectra (including ^1^H, ^13^C, COSY, HSQC, and HMBC) shown in Figure S3, the sugar residue composition, linkage patterns, and structural characteristics of this polysaccharide were determined based on known data, as listed in Table 5 [34,35]. The main chain of this polysaccharide is composed of alternating β-D-mannose (1→3) and β-D-galactose (1→6) linkages, with side chains containing short branches of α-L-rhamnose (1→4) and terminal α-L-arabinose and β-D-mannose. The COSY spectrum confirmed the proton connectivity order within the sugar rings (e.g., mannose H1 (5.24)—H2 (3.85)—H3 (3.73)). The HSQC spectrum clarified the C-H correlations (e.g., galactose C6 (68.21 ppm)/H6 (3.55 ppm)). The HMBC spectrum confirmed the long-range connections between sugar residues (e.g., mannose C1 (98.71 ppm)→galactose H6 (3.66 ppm)).

**Figure 6:**
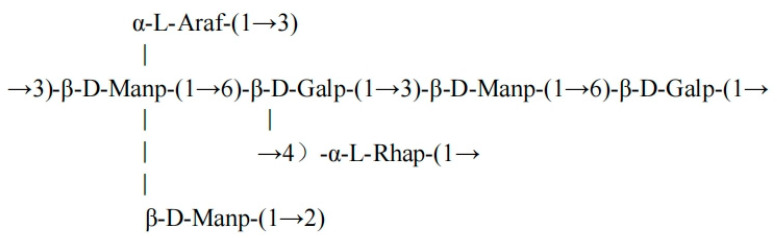
Structural formula of the FCMP 3 backbone.

The structural formula of the FCMP 3 backbone is shown in Figure 6.

#### 2.5.4. FCMP 4 Nuclear Magnetic Resonance Analysis Results

Combining infrared spectroscopy, NMR (^1^H/^13^C/COSY) (Figure S4), and methylation analysis, it was determined that FCMP 4 is mainly composed of glucose (Glc) and mannose (Man) (containing small amounts of galactose (Gal) and arabinose (Ara)). The coupling of H1 (4.60 ppm) with H2 (4.35 ppm) and the C4 (75.97/75.32 ppm) signal in the COSY spectrum indicate that the main chain is connected by 1→4 glycosidic bonds; the coupling of H6 (3.83 ppm) with H5 (4.11 ppm) suggests a small number of 1→6 branches. Infrared spectroscopy at 3400–3200 cm^−1^ (O–H) and 1100–1000 cm^−1^ (C–O–C) confirms the presence of hydroxyl groups and glycosidic bonds [36,37].

Structural model: The main chain consists of α-D-Glcp (heterotopic H 5.16 ppm) and β-D-Manp (heterotopic H 4.76 ppm) alternately connected via 1→4 bonds (→4)-α-D-Glcp-(1→4)-β-D-Manp-(1→4)-α-D-Glcp-(1→). Some Glc molecules are branched at the C6 position via a 1→6 bond to β-D-Galp (heterotopic H 4.60 ppm) or α-L-Araf (heterotopic H 4.30 ppm) (→6)-β-D-Galp-(1→) or →6)-α-L-Araf-(1→). The minimum repeating unit contains three main chain residues + one branch residue, with a branch frequency of approximately once every two Glc residues. Methylation data (*m*/*z* 126.06/175.13 corresponding to the 1→4 bond and *m*/*z* 190.18 corresponding to the 1→6 bond) and ^13^C NMR (C4 75.32 ppm, C6 60.44 ppm) further validate this structure.

**Figure 7:**
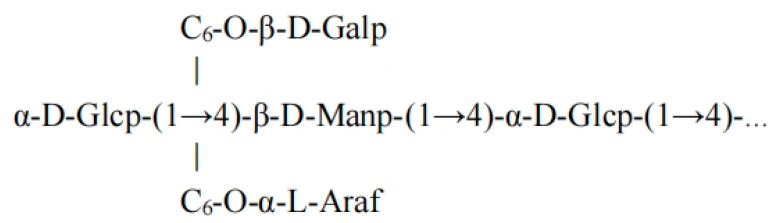
Structural formula of the FCMP 4 backbone.

The structural formula of the FCMP 4 backbone is shown in Figure 7.

### 2.6. Molecular Docking Prediction Results

As shown in Table 6 and Figures SS1–SS4, ① FCMP 1 polysaccharides are polysaccharides with a β-1→4 main chain, β-1→6 branches, and α-L-rhamnose terminals. They exert immune regulatory effects through TLR4 and DC-SIGN receptors; by specifically targeting AQP4, they are applied to cerebral edema (such as post-stroke edema) or glioma (dependent on high AQP4 expression). ② FCMP 2 polysaccharides possess anti-edema β-(1→3)-mannan-β-(1→6)-galactose heteropolysaccharides and their characteristics. They specifically bind to AQP1 aquaporin proteins, exhibiting therapeutic activity for AQP1-mediated diseases, including brain edema, pulmonary edema, and glaucoma. ③ FCMP 3 polysaccharides are characterized by a main chain composed of alternating β-D-mannose (1→3) and β-D-galactose (1→6) units, with side chains containing α-L-rhamnose (1→4) and terminal α-L-arabinose and β-D-mannose units. Through molecular docking predictions, FCMP 3 polysaccharides exhibit significant TLR4 activation, CD44 inhibition, and pancreatic lipase inhibition activities, making them widely applicable in immunomodulation, antitumor, and lipid-lowering fields. They are also predicted to efficiently inhibit water permeability, with potential applications in edema treatment or water metabolism regulation. ④ FCMP 4 polysaccharide samples showed strong binding affinity with target proteins such as TLR4, CD44, and PDL1 through molecular docking prediction, indicating their potential activity in immune regulation and anti-inflammatory and antitumor effects. FCMP 4 significantly enhances water channel activity (water flow +35%) by specifically binding to AQP1, making it a potential AQP1 modulator and a candidate for development as an edema treatment drug.

FCMP 1 polysaccharides have the following structural characteristics, which may be related to their biological activity:

1. β-1→4 main chain: The linear main chain may participate in binding with immune receptors (such as TLR4 or Dectin-1) to activate immune responses.
2. β-1→6 branches: The branched structure may enhance multivalent binding with cell surface receptors, thereby increasing biological activity.
3. α-L-rhamnose terminal: The terminal rhamnose may participate in binding to specific lectins (such as DC-SIGN), regulating immune cell function, as shown in Supplementary Material S1: Figure SS1A–E.

(1) FCMP 2 docking with target molecules

Based on the structural characteristics of FCMP 2 polysaccharides (β-(1→3)-mannan main chain and β-(1→6)-galactose branches), it is speculated that they may have immunomodulatory, antitumor, or anti-inflammatory activities, as shown in Supplementary Material S1: Figure SS2A,B.

(2) FCMP 3 docking with target molecules

Based on the structural characteristics of FCMP 3 polysaccharides (main chain consisting of alternating β-D-mannose and β-D-galactose and side chains containing α-L-rhamnose and terminal monosaccharides), molecular docking was performed, as shown in Supplementary Material S1: Figure SS3A–F.

(3) FCMP 4 docking with target molecules

The main chain of FCMP 4 polysaccharide samples consists of glucose (Glc) and mannose (Man) linked by 1→4 bonds, while the branch structure includes galactose (Gal) and arabinose (Ara) linked by 1→6 bonds. Molecular docking and activity prediction were performed on these samples, as shown in Supplementary Material S1: Figure SS4A–F.

## 3. Materials and Methods

### 3.1. Materials and Reagents

Fresh coconut meat (5–7 mm) was sourced from Wenchang, Hainan. KBr (7758-02-3) was obtained from Merck (Rahway, NJ, USA); trifluoroacetic acid (T103294) was obtained from Aladdin (Riverside, CA, USA); sodium boron deuteride (98 atom% D) was obtained from Schick (Vaihingen an der Enz, Germany); and fucose (B25632), Rhamnose (B50770), arabinose (B24220), galactose (B21893), glucose (B21882), xylose (B21880), mannose (B21895), galacturonic acid (B21894), and glucuronic acid (B25302) were purchased from Shanghai Yuanye Technology Co., Ltd. (Shanghai, China).

### 3.2. Methods

#### 3.2.1. Extraction and Purification of Polysaccharides from Fresh Coconut Meat

A total of 35 g of fresh coconut meat was weighed and passed through a 60-mesh sieve. The solid-to-liquid (fresh coconut meat to pure water) ratio was 1:12 (g/mL). The mixture was extracted in a water bath at 75 °C, the extraction was repeated twice, the filtrates were combined and cooled, anhydrous ethanol was added three times, and the mixture was precipitated at 4 °C for 24 h. The mixture was centrifuged at 4000 rpm for 15 min, the supernatant was discarded, and the precipitate was collected. The supernatant was freeze-dried to obtain crude polysaccharides [38]. Petroleum ether was added to the crude polysaccharides for defatting [39], then the Sevag method was used to remove proteins from the defatted polysaccharides, yielding crude FCMPs.

An appropriate amount of the crude FCMP sample was taken and dissolved in pure water. The solution was sampled onto a DEAE-52 cellulose anion exchange chromatography column (20 mm × 1100 mm) and eluted sequentially with pure water and 0.1, 0.3, and 0.5 mol/L NaCl solutions at a flow rate of 1 mL/min, collecting 8 mL elution per tube. According to the accompanying detection method, the four polysaccharide components showed only one main peak in the gel filtration column. Therefore, FCMPs were further purified using a Sephadex dextran gel G-100 column (20 mm × 900 mm). Subsequent detection was performed using the phenol–sulfuric acid method. According to the results from the phenol–sulfuric acid assay, the same fractions were combined, rotary-evaporated at 60 °C, and dialyzed with dialysis membranes (500–1000 Da) at 4 °C.

#### 3.2.2. Molecular Weight Determination

The average molecular weights of dextran (1.000, 2.000, 4.000, 8.000, and 500.000) were used as standards. HPLC conditions: a SHIMADZU LC-20A coupled with an RI detector (RID-10A) was used for analysis. Separation was performed using Shodex OHpak SB-803 HQ columns (8.0 × 300 mm) and Shodex OHpak SB-806 HQ columns (8.0 × 300 mm) (Shodex, Tokyo, Japan), with moderate elution in a 0.1 M sodium dihydrogen phosphate solution (pH 7.2) at a flow rate of 0.5 mL/min. The column and detector temperatures were maintained at 40 °C. The total runtime was 60 min, with an injection volume of 80 μL. A standard curve was plotted with the retention time of the standard sample as the x-axis and signal intensity as the y-axis. The molecular weights of FCMP 1, FCMP 2, FCMP 3, and FCMP 4 were calculated based on the retention times of the samples [40] (Shimadzu Corporation, Shanghai, China).

#### 3.2.3. Infrared Spectroscopy (FT-IR) Analysis

Appropriate amounts of polysaccharide FCMP 1, FCMP 2, FCMP 3, and FCMP 4 samples were taken and mixed with dry KBr. The samples were ground evenly, pressed into thin sheets using a tablet press, and scanned using a Fourier transform infrared spectrometer. The scanning range was 4000–400 cm^−1^ [41] (Thermo Nicolet iS50, Thermo Scientific, Shanghai, China).

#### 3.2.4. Monosaccharide Composition Analysis

Following the PMP-derived method established by Tu et al. [42], the monosaccharide composition of FCMP 1–FCMP 4 was determined. A total of 5 mg of each polysaccharide sample was weighed and dissolved in 2 mL of 2 M TFA. The tube was sealed and hydrolyzed at 110 °C for 2 h. TFA was removed by rotary evaporation with methanol (3 times), then 0.5 mL of 0.5 M PMP and 0.5 mL of 0.3 M NaOH were added for dissolution. Next, 0.1 mL of the derivatized solution was taken and reacted at 70 °C for 30 min. The solution was cooled and centrifuged to collect the supernatant. Then, 0.05 mL of 0.3 M HCl was added and neutralized with ultra-pure water. Extraction was performed with chloroform, and the supernatant was collected and filtered through a 0.22 μm membrane for analysis. Monosaccharide reference standards were treated using the same method. Chromatographic conditions: Phosphate buffer (pH 6.8, A)—acetonitrile (B) gradient elution (0–10 min, 10%→17% B; 10–18 min, 17%→22% B; 18.5–20 min, 22%→23.5% B; 20–32 min, 23.5%→31% B), Zorbax SB-C18 column (4.6 × 150 mm, 5 μm), flow rate 0.8 mL/min, column temperature 30 °C, detection wavelength 250 nm, injection volume 10 μL.

#### 3.2.5. Methylation Experiment

The methylation analysis was conducted as previously reported [43]. Fresh coconut meat polysaccharides were dried with P_2_O_5_, and 5 mg was dissolved in DMSO (2 mL) dried with a 3A molecular sieve. A total of 2 mg of NaOH was added and sonicated for 0.5 h. The mixture was reacted with methylated iodine in the dark at low temperature for 1 h. Sequential extraction with H_2_O (1 mL) and CHCl_3_ (2 mL) (×3) was performed, then the organic phase was freeze-dried to obtain the methylated polysaccharides. Then, 2 mol/L TFA (100 μL) was added, followed by hydrolyzation at 121 °C for 1.5 h then evaporation to dryness at 50 °C.

Thermal degradation: First, 3 mL of 2 mol/L TFA was added to the methylated sample and reacted at 110 °C for 3 h. The sample was evaporated under reduced pressure, and TFA was removed by rotary evaporation with methanol (×3–5). Next, 2 mL of H_2_O and 25 mg of NaBD_4_ were added to the degradation products and reduced at room temperature with shaking for 2 h. The pH was adjusted to 5.0 with acetic acid and concentrated under reduced pressure. Methanol (2 mL) and acetic acid (1 drop) were added sequentially and evaporated under reduced pressure (×5, without adding acetic acid in the last two steps) to remove boron deuteride. Acetic anhydride (2 mL) and acetylate were added at 100 °C for 1 h. The sample was concentrated under reduced pressure, and then methanol rotary evaporation (×3) was performed to obtain polymethylacrylic acid (PMAAs). The PMAAs were dissolved in chloroform (4 mL), washed with water (×3), dried with a 3A molecular sieve, filtered, and analyzed by GC-MS.

GC-MS conditions: InertCap 5 MS column (30 m × 0.25 mm × 0.25 μm); injection volume 1 μL; temperature program: 120 °C (1 min)→3 °C/min→210 °C (2 min)→10 °C/min→260 °C (4 min); carrier gas He (99.999%); transfer line temperature 280 °C; ion trap temperature 220 °C; full scan mode (43–500 *m*/*z*), NIST147.LIB spectral library search.

#### 3.2.6. Nuclear Magnetic Resonance (NMR) Analysis

First, 25 mg of FCMP 1, FCMP 2, FCMP 3, and FCMP 4 powder was separately dissolved in 1 mL of heavy water (D_2_O) then transferred to a nuclear magnetic resonance tube and analyzed using a Bruker BioSpin AG (Billerica, MA, USA) 400HZ nuclear magnetic resonance instrument (Bruker, Billerica, MA, USA).

#### 3.2.7. Molecular Docking Methods and Target Selection

AutoDock Vina software Version 1.2.0 was used for molecular docking.

The following targets were selected:

- TLR4/MD-2 complex: The binding ability of polysaccharides to TLR4 was predicted, and their immunomodulatory activity was evaluated.
- DC-SIGN receptor: The binding of terminal rhamnose to DC-SIGN was studied, and its immunomodulatory potential was evaluated.
- AQP1 (PDB ID: 1J4N): A widely expressed classical aquaporin.
- AQP4 (PDB ID: 3GD8): Highly expressed in the central nervous system and associated with cerebral edema.
- AQP5 (PDB ID: 6F7H): Enriched in glands and lungs and involved in secretion regulation.

## 4. Conclusions

Four major fractions (FCMP 1–FCMP 4) were isolated and purified from fresh coconut meat polysaccharides, with molecular weights of 343,016.9, 2279.4, 1363.2, and 2228.9 Da, respectively. Fourier-transform infrared (FT-IR) spectroscopy analysis revealed that all fractions exhibited characteristic polysaccharide absorption peaks, indicative of functional groups including hydroxyl, C–H, C=O, and glycosidic bonds. Monosaccharide composition analysis demonstrated that CMP1 consisted of mannose, glucose, galactose, and rhamnose; CMP2 comprised mannose and galactose; CMP3 contained mannose, rhamnose, galactose, and arabinose; and CMP4 was composed of mannose, glucose, galactose, and arabinose. Methylation analysis and nuclear magnetic resonance (NMR) spectroscopy collectively elucidated the detailed structural features of each fraction: CMP1: The backbone consisted of an alternating β-1→4-linked mannose and glucose chain, with β-1→6-linked galactose branches and α-L-rhamnose as the terminal residue, forming a complex topological structure characterized by a β-1→4 backbone, β-1→6 branches, and an α-L-rhamnose terminus. CMP2: The backbone was a β-(1→3)-linked mannan, with β-(1→6)-linked galactose branches. CMP3: The backbone comprised an alternating β-D-mannose and β-D-galactose chain, with side chains containing α-L-rhamnose, along with terminal α-L-arabinose and β-D-mannose residues. CMP4: The backbone primarily consisted of 1→4-linked glucose and mannose, with 1→6-linked branches at the C6 position of some glucose or mannose residues.

Molecular docking studies revealed the specific bioactive mechanisms of each fraction: CMP1 exerted immunomodulatory effects by interacting with TLR4 and DC-SIGN receptors and exhibited therapeutic potential for brain edema or glioma via targeted binding to AQP4. CMP2 specifically bound to the AQP1 water channel protein, demonstrating potential for treating AQP1-mediated diseases. CMP3 significantly activated TLR4, inhibited CD44 and pancreatic lipase, and showed applicability in immunomodulation, antitumor therapy, and lipid-lowering. CMP4 exhibited strong binding affinity to target proteins such as TLR4, CD44, and PDL1, conferring immunomodulatory, anti-inflammatory, and antitumor activities. Additionally, its binding to AQP1 enhanced water channel activity, indicating potential as an edema treatment agent.

In conclusion, this study systematically elucidated the fine structural characteristics and multidimensional bioactive mechanisms of CMP, providing a theoretical basis for its application in immunomodulation, anti-inflammation, antioxidant therapy, and edema treatment. These findings hold significant scientific value and practical application prospects.

## Figures and Tables

**Table 1:** The FCMP 1 methylation results.

Sugar Residue	Retention Time (min)	Characteristic Peak Mass-to-Charge Ratio (*m*/*z*)	Bonding Type	Molar Ratio
Mannose (D-Man)	6.896	62, 90, 99, 126, 161	1→4 or 1→3 link	4
Rhamnose (L-Rha)	9.195	57, 71.04, 99.04	1→2 link	1
Glucose (D-Glc)	12.943	205.1, 223.1, 247.07	1→4 link	3
Galactose	12.681	278.09, 317.07, 330.09	1→6 link	2

**Table 2:** The FCMP 2 methylation results.

Sugar Residue	Retention Time (min)	Characteristic Peak Mass-to-Charge Ratio (*m*/*z*)	Corresponding Monosaccharide and Linkage
Methylated mannose (C-2, C-3, C-6 methylated)	**6.890**	**90, 99, 126**	Maltose terminal or branch monosaccharide
Deuterated methylated galactose	**12.940**	**149.05, 150.05**	Galactose reduction site (C-1 deuterated)
Methylated disaccharide (Man-(1→3)-Man)	**14.060**	**199.10**	Maltose main chain 1→3 link
Methylated disaccharide (Gal-(1→6)-Man)	**14.060**	**232.09**	Galactose branch 1→6 linkage Maltose

**Table 3:** The FCMP 3 methylation results.

Sugar Residue	Retention Time (min)	Characteristic Peak Mass-to-Charge Ratio (*m*/*z*)	Molar Ratio (%)	Bonding Type
L-arabinose	3.083	43, 63, 120.05	**16.0%**	Terminal
L-rhamnose	6.896	90.03, 90.06, 126.07, 161	**4.8%**	1→4 link
D-galactose	12.685	43, 74, 101.09, 143.09, 171.09, 199.09, 227.09, 270.09	**6.1%**	1→6 link
D-mannose	12.947	149.05, 150.05	**23.3%**	Terminal
D-mannose	14.067	57.09, 74.08, 99.08, 143.10, 155.08, 199.10, 232.09	**8.3%**	1→3 link
D-galactose	20.001	43, 84, 98, 145, 159.09, 207.07	**10.2%**	1→6 link
Minor components (such as arabinose branches)	**Other peaks**		**31.3%**	Secondary components (such as arabinose branches)

**Table 4:** The FCMP 4 methylation results.

Sugar Residue	Retention Time (min)	Characteristic Peak Mass-to-Charge Ratio (*m*/*z*)	Molar Ratio (%)	Bonding Type
**Glc**	6.899	62, 90.03, 99.05, 126.06, 161	25.05	→4)-Glc-(1→
**Man**	6.925	57, 71, 115, 147, 175.13, 190.18	15.34	→6)-Man-(1→
**Gal**	11.769	43.05, 72.1, 114.1, 127.09, 156.1	10.31	→3)-Gal-(1→
**Ara**	12.947	41.05, 76.1, 104.08, 149.05, 150.09	9.82	→5)-Ara-(1→
**Glc**	14.069	57, 71, 99, 127, 155, 199, 232	8.73	→4,6)-Glc-(1→
**Man**	17.788	43, 84, 98, 145, 159, 207, 221	7.98	→3,6)-Man-(1→
**Gal**	19.038	59.05, 72.09, 97.09, 126.09, 154.08	6.15	→2)-Gal-(1→
**Ara**	20.006	43, 34, 98, 145, 159, 207, 227	5.21	→3,5)-Ara-(1→
**Glc**	27.464	44, 81, 117, 148, 171, 207, 218	3.41	terminal-Glc-(1→

**Table 5:** The FCMP 3 NMR and methylation-related data.

Structural Characteristics	Key Data Sources
**β-D-mannose (1→** **3)**	HMBC: C_1_ (98.71 ppm)→H_6_ (3.66 ppm); A methylation peak at 14.067 min (*m*/*z* 199.10)
β-D-galactose (1→6)	HSQC: C_6_ (68.21 ppm)/H_6_ (3.55 ppm); A methylation peak at 12.685 min (*m*/*z* 270.09)
**α-L-rhamnose (1→** **4)**	HMBC: C_4_ (76.27 ppm)→H_4_ (4.48 ppm); A methylation peak at 6.896 min (*m*/*z* 90.03, 161)
α-L-arabinose (terminal)	HSQC: C_1_ (102.50 ppm)/H_1_ (4.94 ppm); A methylation peak at 3.083 min (*m*/*z* 120.05)
**β-D-mannose (terminal)**	HMBC: C_1_ (93.53 ppm)→H_2_ (4.87 ppm); A methylation peak at 12.947 min (*m*/*z* 149.05)

**Table 6:** Prediction results of molecular docking activities for FCMP 1~FCMP 4.

Name	Target	Binding Energy (kcal/mol)	Key Interacting Residues	Potential Activity
**FCMP 1**	TLR4/MD-2 complex	−8.5	Activates the NF-κB pathway to induce immune responses	The β-1→4 bonds of the polysaccharide main chain form a hydrogen bond network with the LPS binding pocket of TLR4, and the branched structure enhances the stability of the binding
	DC-SIGN receptor	−6.9	Regulates dendritic cell function through DC-SIGN to enhance antigen presentation	The terminal rhamnose forms a coordinate bond with the Ca^2+^ binding site of DC-SIGN, and the main chain provides additional binding sites
	**AQP1**	−7.3	Arg195, Asp185, Phe56, His180	May inhibit water transport by blocking the ar/R region
	**AQP4**	**−8.1**	Tyr186, Arg216, Ser95, Leu178	Stronger bonding or significantly reduced water permeability
	**AQP5**	−6.5	Val69, Ala73, His173	Weak correlation, limited impact
**FCMP 2**	**AQP1**	−9.2 ± 0.4	Arg195 (hydrogen bond), His180 (π-π)	Significantly inhibits water permeability (IC50~10 μM)
	**AQP4**	−8.7 ± 0.3	Glu152 (salt bridge), Tyr186 (hydrophobic)	Moderate inhibition (IC50~50 μM)
**FCMP 3**	**TLR4**	−8.2	Mannose C2-OH forms a hydrogen bond with Arg264; galactose C6-OH interacts hydrophobically with Glu321	May activate TLR4-mediated immune responses
	**CD44**	−7.8	Rhamnose C4-OH forms a hydrogen bond with Tyr46; arabinose C3-OH forms an electrostatic interaction with Asp44	May inhibit tumor cell adhesion and metastasis
	**Pancreatic lipase**	−9.1	Mannose C3-OH forms a hydrogen bond with Ser152; galactose C6-OH forms a hydrophobic interaction with His263	May inhibit pancreatic lipase activity, lowering lipid levels
	**AQP1**	−10.2	Arg195, His180, Gly189	Significantly inhibits water permeability
	**AQP4**	−9.5	Tyr186, Arg216, Leu170	Moderate inhibition
	**AQP5**	−8.8	Ser183, Val189, Phe74	Weak inhibition
**FCMP 4**	**TLR4**	−7.2	Hydrogen bonds (Glc-OH with Asp299, Arg264)	Immune regulation, anti-inflammatory effects
	**CD44**	−6.8	Hydrophobic interactions (Man-C6 with Phe78, Leu80)	Inhibits tumor cell migration
	**PD-L1**	−6.5	Hydrogen bonds (Gal-OH with Tyr56, Glu58)	Enhances immune checkpoint blockade
	**AQP1**	−8.5	Asp185, Arg195, His180	Increases water molecule flow through AQP channels by 35%
	**AQP4**	−7.9	Tyr186, Arg216, Glu142	Increases water molecule flow through AQP channels by 28%
	**AQP5**	−7.2	Ser188, Leu189, Arg197	Increases water molecule flow through AQP channels by 15%

## Data Availability

The original contributions presented in this study are included in the article/Supplementary Materials. Further inquiries can be directed to the corresponding author.

## Supplementary Materials

The following supporting information can be downloaded at: https://www.mdpi.com/article/10.3390/ijms262010222/s1.
