# Supplementary material for: Structural Characterization and Molecular Docking Studies of Fresh Coconut Meat Polysaccharides
Source: Int J Mol Sci. 2025 Oct 21;26(20):10222. doi: 10.3390/ijms262010222 (PMC12564216; doi:10.3390/ijms262010222)
Supplement: Supplementary file 1 [file ijms-26-10222-s001.zip › ijms-3761793-supplementary.pdf]

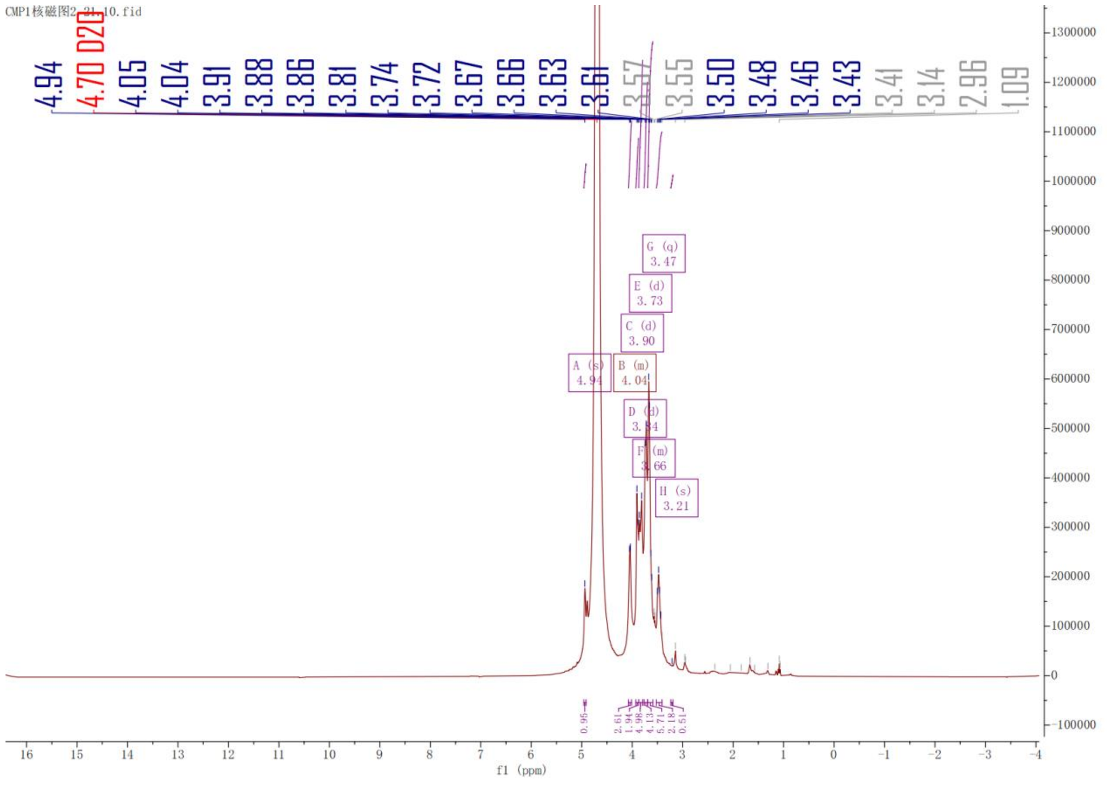
CMP1核磁图2.21.10.fid
4.94
4.70 D2O
4.05
4.04
3.91
3.88
3.86
3.81
3.74
3.72
3.67
3.66
3.63
3.61
3.57
3.55
3.50
3.48
3.46
3.43
3.41
3.14
2.96
1.09
A (s) 4.94
B (m) 4.04
C (d) 3.90
D (d) 3.84
E (d) 3.73
F (m) 3.66
G (q) 3.47
H (s) 3.21
f1 (ppm)

(**a**)

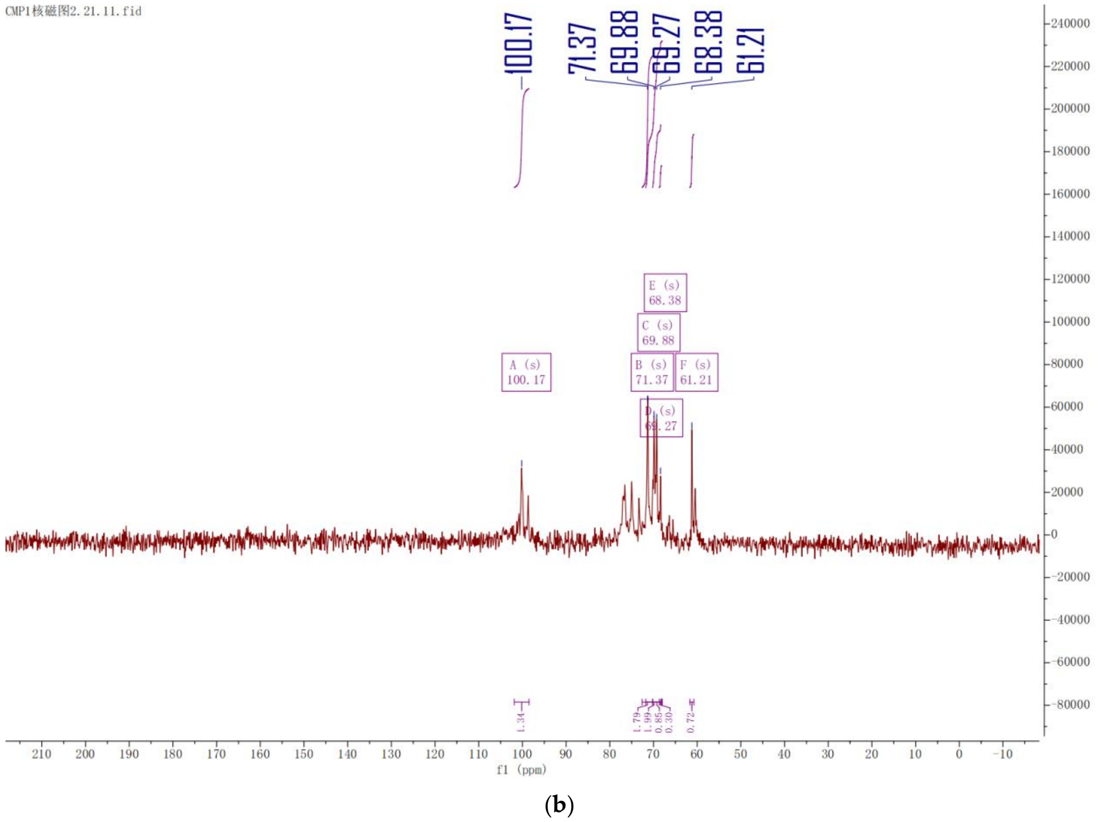
CMP1核磁图2.21.11.fid
100.17
71.37
69.88
69.27
68.38
61.21
A (s) 100.17
B (s) 71.37
C (s) 69.88
D (s) 69.27
E (s) 68.38
F (s) 61.21
f1 (ppm)

(**b**)

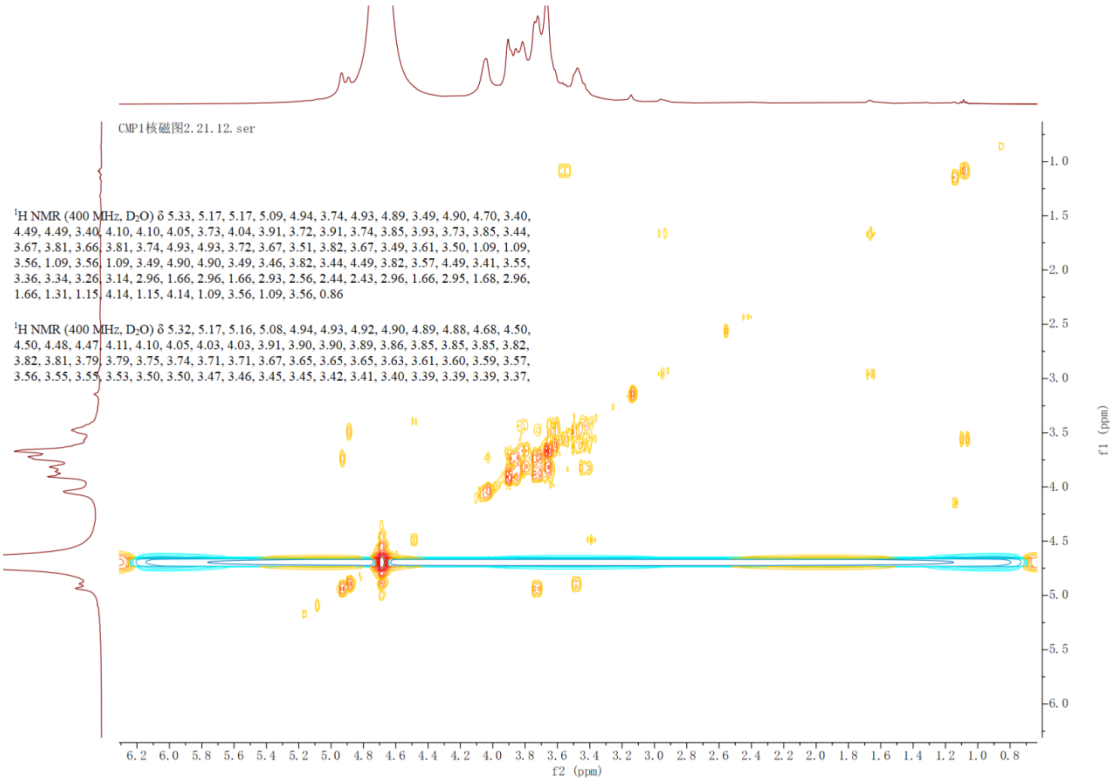
CMP1核磁图2.21.12.ser
¹H NMR (400 MHz, D₂O) δ 5.33, 5.17, 5.17, 5.09, 4.94, 3.74, 4.93, 4.89, 3.49, 4.90, 4.70, 3.40, 4.49, 4.49, 3.40, 4.10, 4.10, 4.05, 3.73, 4.04, 3.91, 3.72, 3.91, 3.74, 3.85, 3.93, 3.73, 3.85, 3.44, 3.67, 3.81, 3.66, 3.81, 3.74, 4.93, 4.93, 3.72, 3.67, 3.51, 3.82, 3.67, 3.49, 3.61, 3.50, 1.09, 1.09, 3.56, 1.09, 3.56, 1.09, 3.49, 4.90, 4.90, 3.49, 3.46, 3.82, 3.44, 4.49, 3.82, 3.57, 4.49, 3.41, 3.55, 3.36, 3.34, 3.26, 3.14, 2.96, 1.66, 2.96, 1.66, 2.93, 2.56, 2.44, 2.43, 2.96, 1.66, 2.95, 1.68, 2.96, 1.66, 1.31, 1.15, 4.14, 1.15, 4.14, 1.09, 3.56, 1.09, 3.56, 0.86
¹H NMR (400 MHz, D₂O) δ 5.32, 5.17, 5.16, 5.08, 4.94, 4.93, 4.92, 4.90, 4.89, 4.88, 4.68, 4.50, 4.50, 4.48, 4.47, 4.11, 4.10, 4.05, 4.03, 4.03, 3.91, 3.90, 3.90, 3.89, 3.86, 3.85, 3.85, 3.85, 3.82, 3.82, 3.81, 3.79, 3.79, 3.75, 3.74, 3.71, 3.71, 3.67, 3.65, 3.65, 3.65, 3.63, 3.61, 3.60, 3.59, 3.57, 3.56, 3.55, 3.55, 3.53, 3.50, 3.50, 3.47, 3.46, 3.45, 3.45, 3.42, 3.41, 3.40, 3.39, 3.39, 3.39, 3.37,
f2 (ppm)
f1 (ppm)

(c)

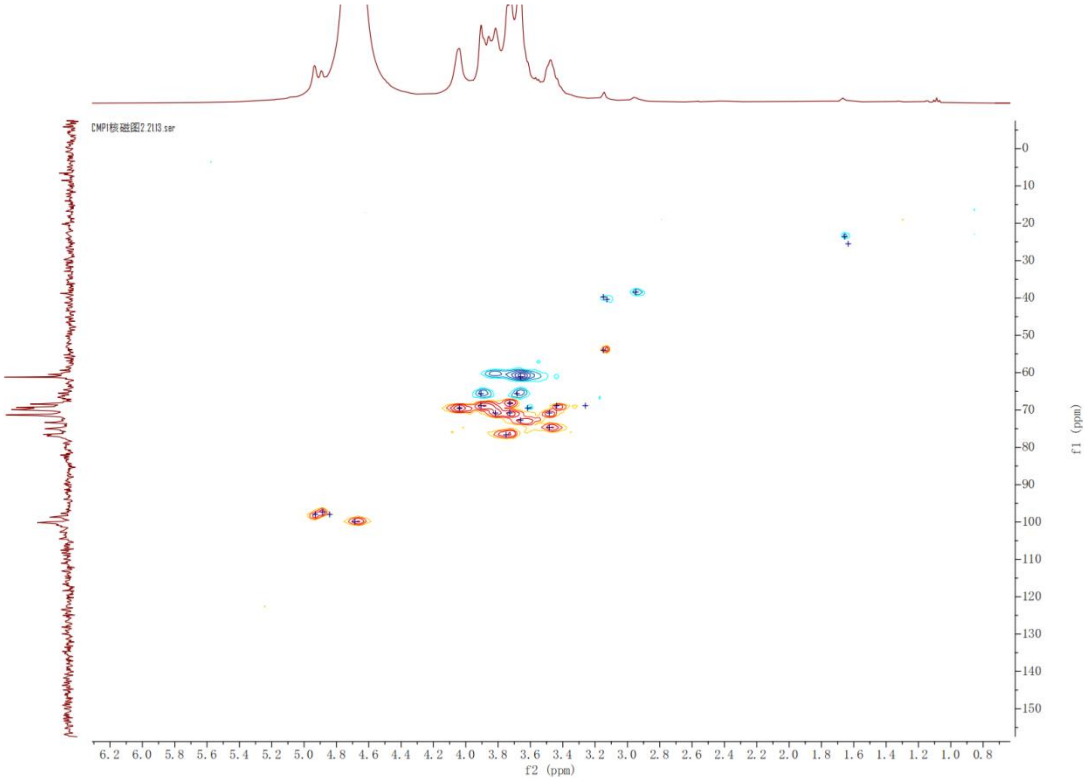
CMP1核磁图2.2113.ser
f2 (ppm)
f1 (ppm)

(d)

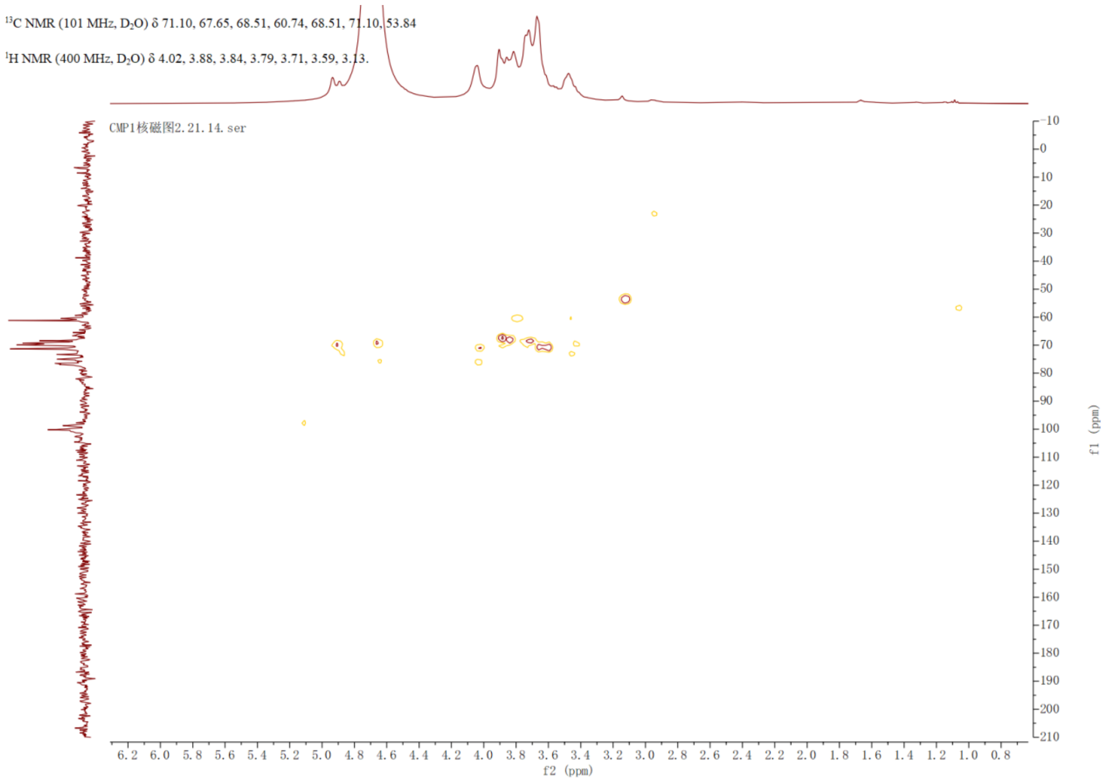

(e)

Figure S1. NMR analysis of FCMP 1: (a) ^{13}C NMR (b) ^{1}H NMR (c) COSY (d) HSQC (e) HMBC.

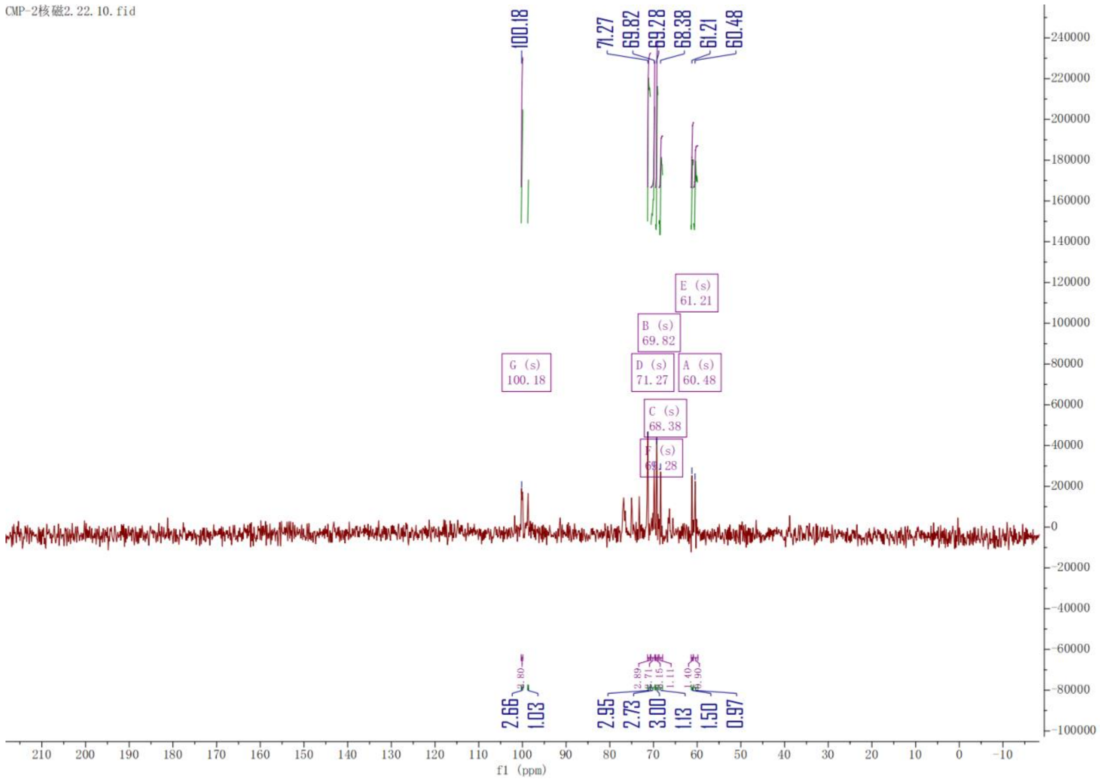
CMP-2核磁2.22.10.fid
100.18
71.27
69.82
69.28
68.38
61.21
60.48
G (s) 100.18
D (s) 71.27
B (s) 69.82
C (s) 68.38
F (s) 69.28
E (s) 61.21
A (s) 60.48
f1 (ppm)

(a)

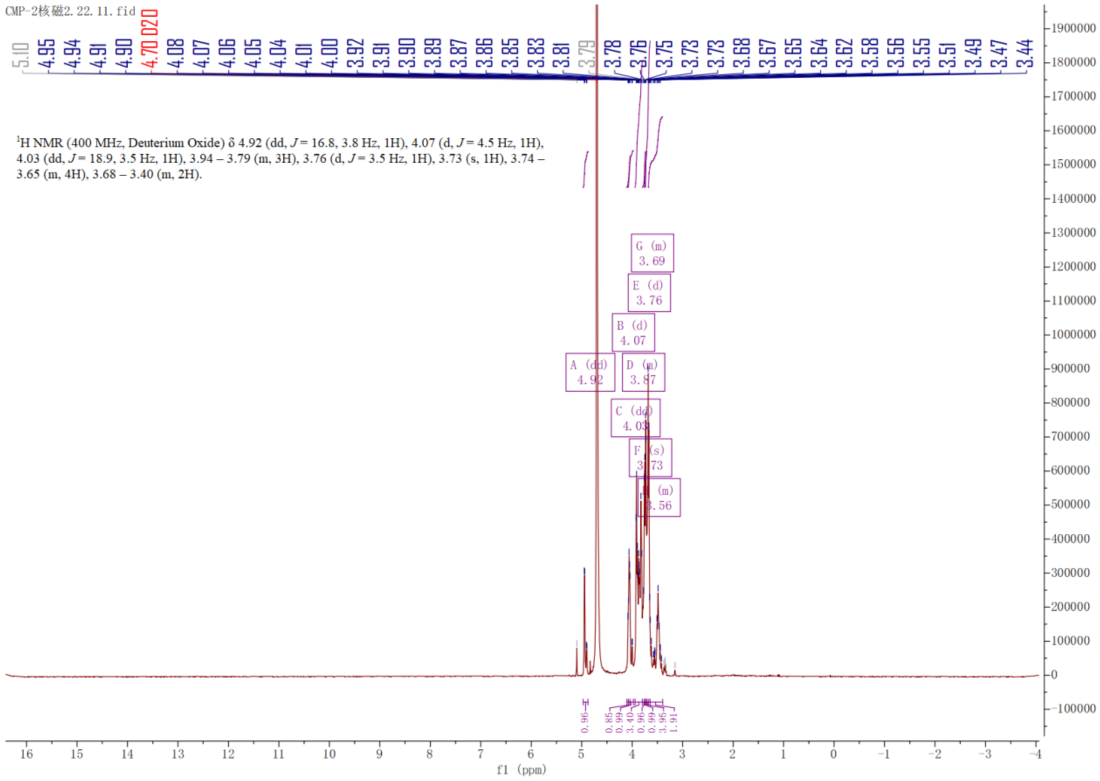
CMP-2核磁2.22.11.fid
4.70 D2O
^{1}H NMR (400 MHz, Deuterium Oxide) δ 4.92 (dd, J = 16.8, 3.8 Hz, 1H), 4.07 (d, J = 4.5 Hz, 1H), 4.03 (dd, J = 18.9, 3.5 Hz, 1H), 3.94 – 3.79 (m, 3H), 3.76 (d, J = 3.5 Hz, 1H), 3.73 (s, 1H), 3.74 – 3.65 (m, 4H), 3.68 – 3.40 (m, 2H).
A (dd) 4.92
B (d) 4.07
C (dd) 4.03
D (m) 3.87
E (d) 3.76
F (s) 3.73
G (m) 3.69
f1 (ppm)

(b)

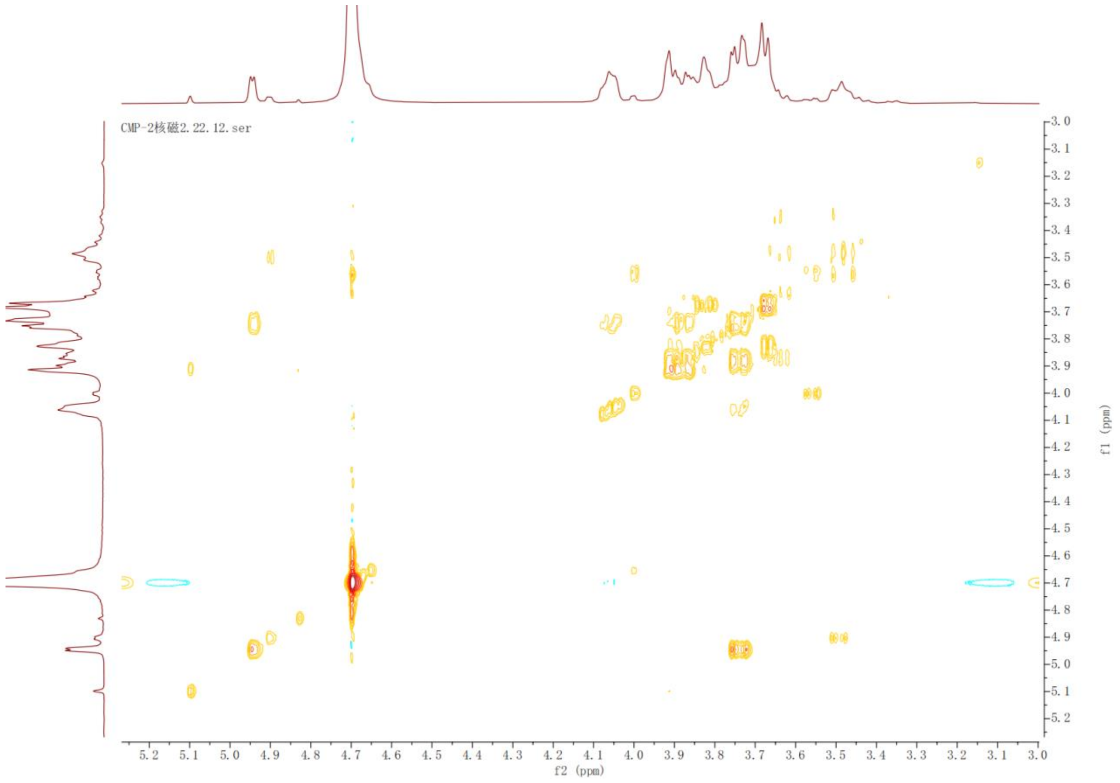

(c)

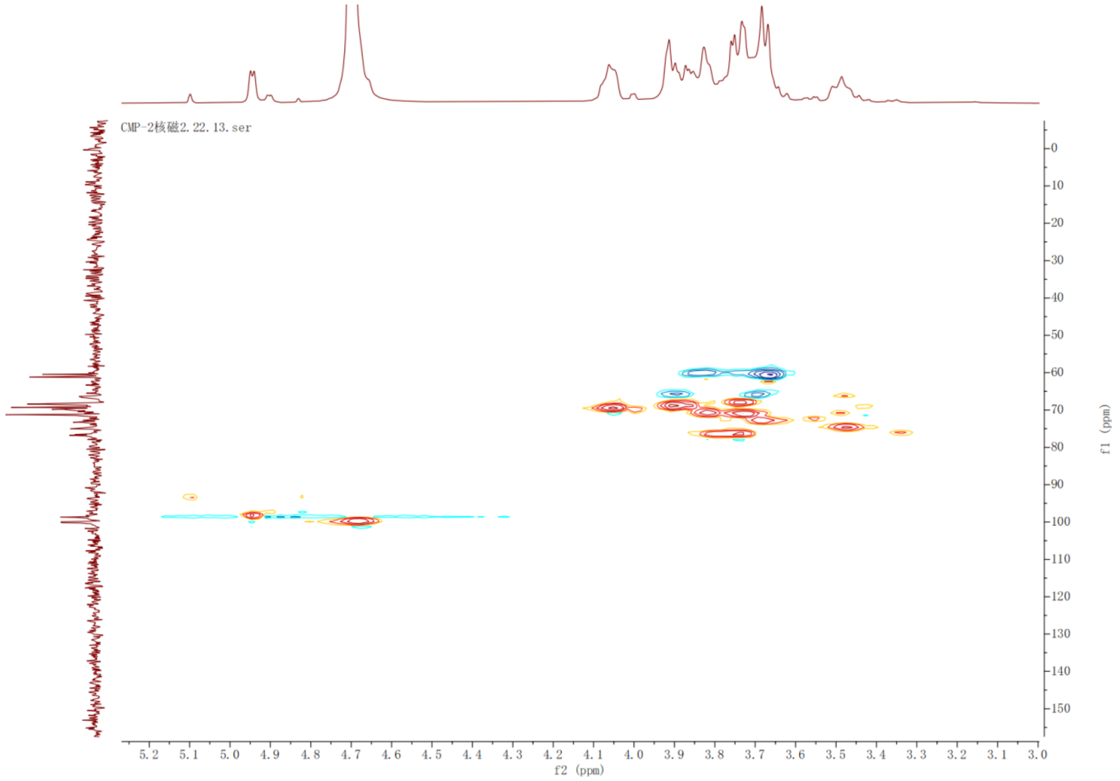

(d)

Figure S2. NMR analysis of FCMP 2: (a) ^{13}C NMR, (b) ^{1}H NMR, (c) COSY, (d) HSQC.

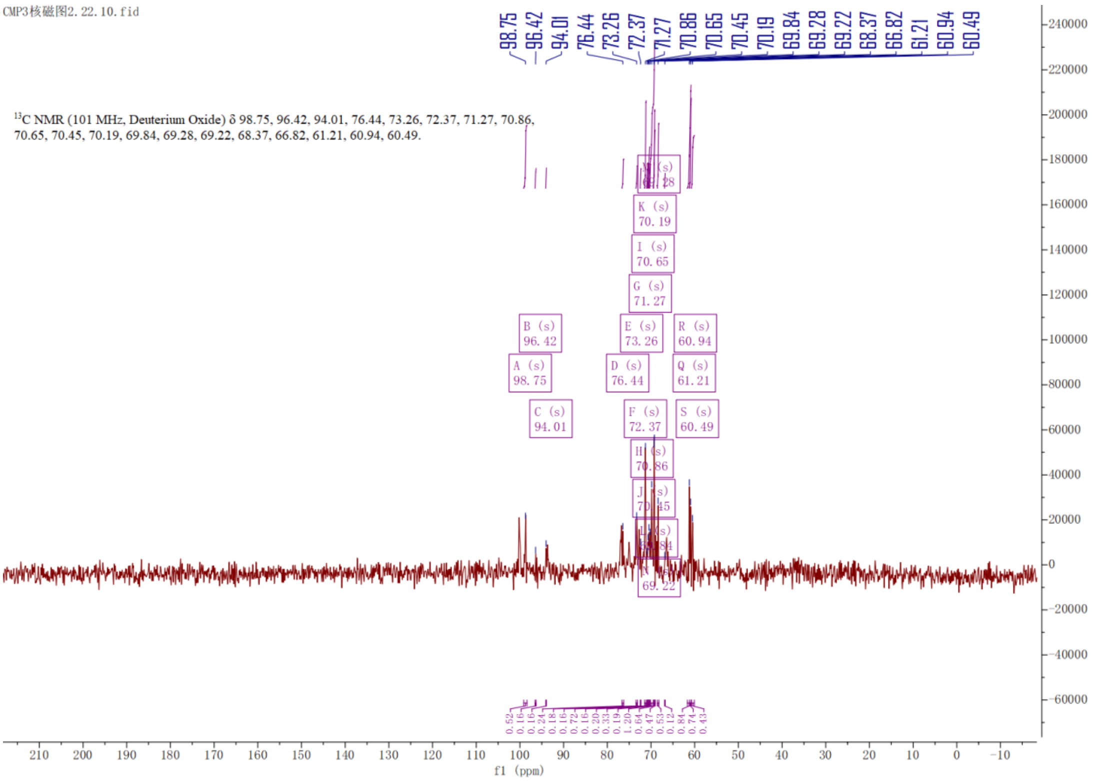
CMP3核磁图2.22.10.fid
13C NMR (101 MHz, Deuterium Oxide) δ 98.75, 96.42, 94.01, 76.44, 73.26, 72.37, 71.27, 70.86, 70.65, 70.45, 70.19, 69.84, 69.28, 69.22, 68.37, 66.82, 61.21, 60.94, 60.49.
f1 (ppm)

(a)

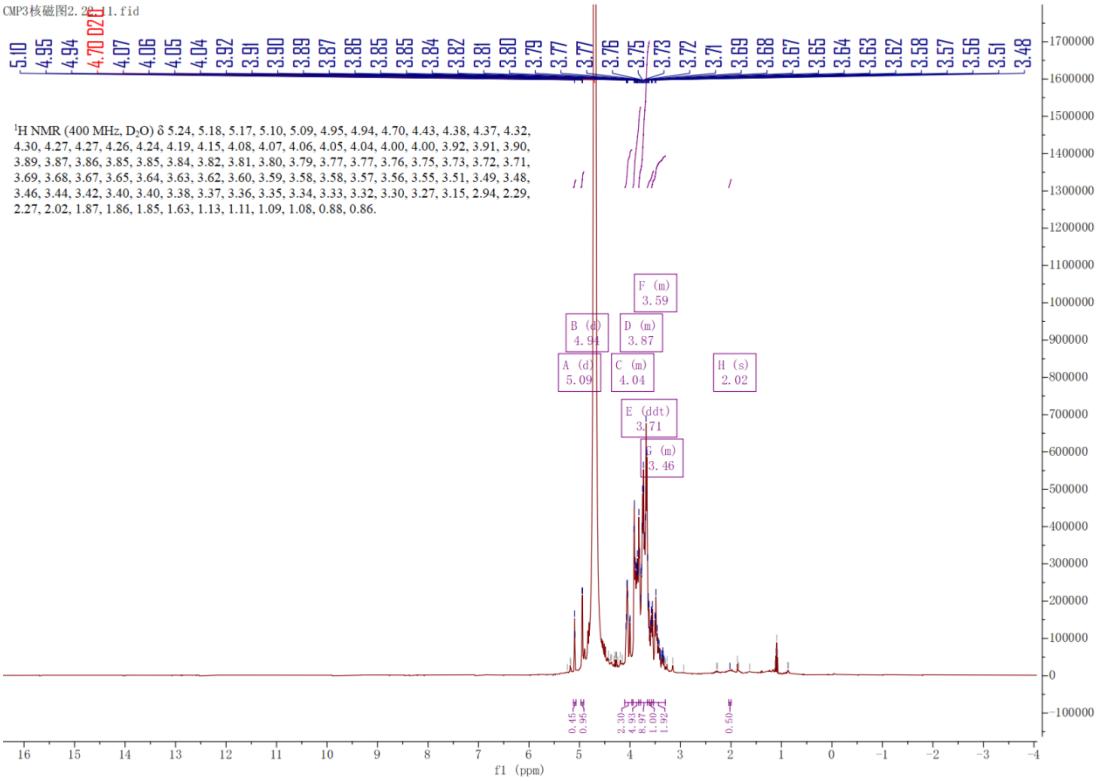
1H NMR (400 MHz, D2O) δ 5.24, 5.18, 5.17, 5.10, 5.09, 4.95, 4.94, 4.70, 4.43, 4.38, 4.37, 4.32, 4.30, 4.27, 4.27, 4.26, 4.24, 4.19, 4.15, 4.08, 4.07, 4.06, 4.05, 4.04, 4.00, 4.00, 3.92, 3.91, 3.90, 3.89, 3.87, 3.86, 3.85, 3.85, 3.84, 3.82, 3.81, 3.80, 3.79, 3.77, 3.77, 3.76, 3.75, 3.73, 3.72, 3.71, 3.69, 3.68, 3.67, 3.65, 3.64, 3.63, 3.62, 3.60, 3.59, 3.58, 3.58, 3.57, 3.56, 3.55, 3.51, 3.49, 3.48, 3.46, 3.44, 3.42, 3.40, 3.40, 3.38, 3.37, 3.36, 3.35, 3.34, 3.33, 3.32, 3.30, 3.27, 3.15, 2.94, 2.29, 2.27, 2.02, 1.87, 1.86, 1.85, 1.63, 1.13, 1.11, 1.09, 1.08, 0.88, 0.86.
4.70 D2O
f1 (ppm)

(b)

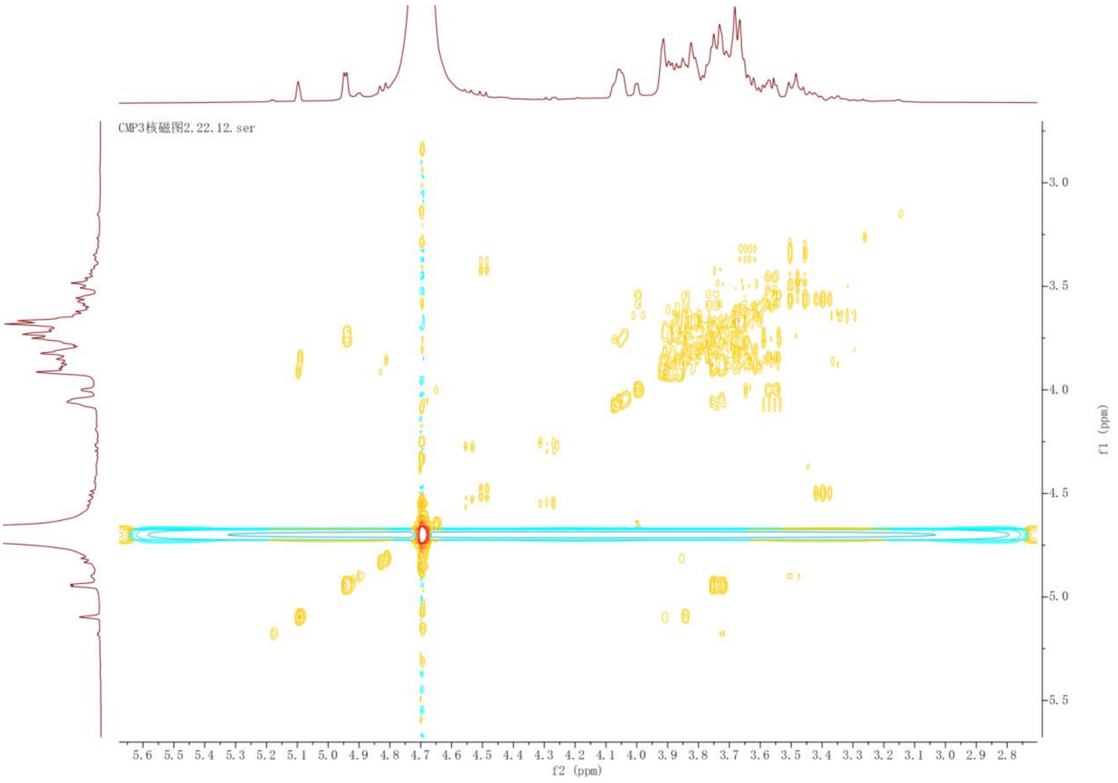
CMP3核磁图2.22.12.ser
f2 (ppm)
f1 (ppm)

(c)

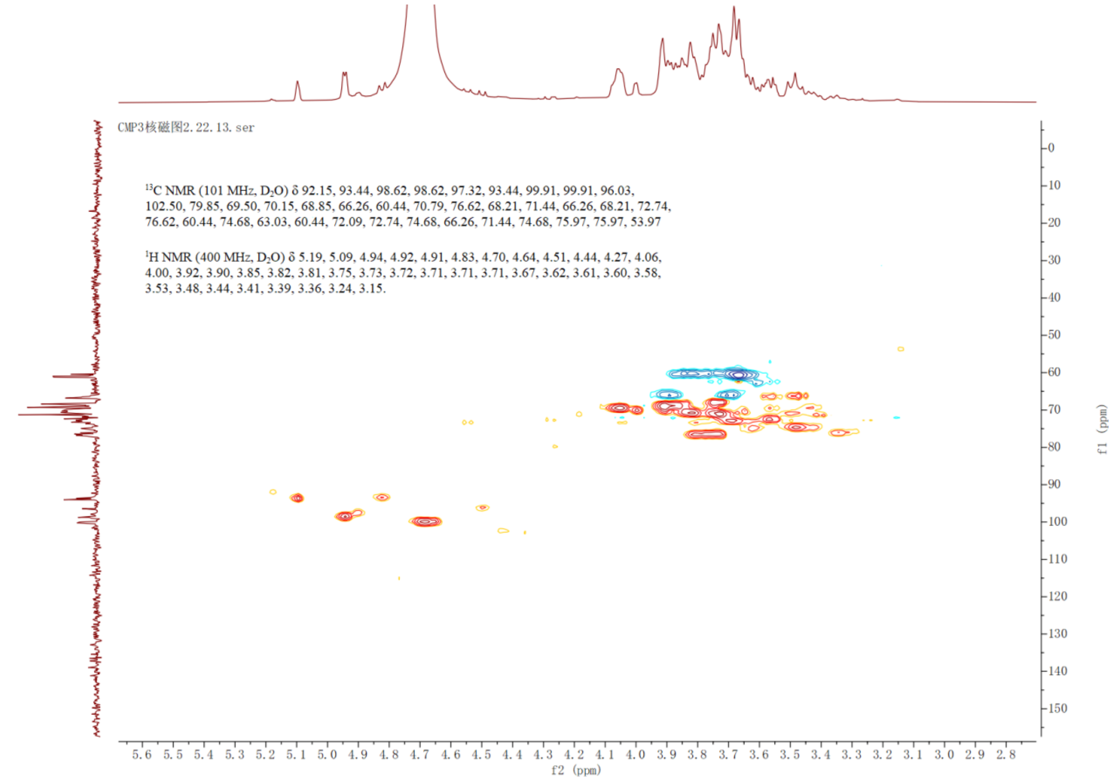
CMP3核磁图2.22.13.ser
13C NMR (101 MHz, D2O) δ 92.15, 93.44, 98.62, 98.62, 97.32, 93.44, 99.91, 99.91, 96.03, 102.50, 79.85, 69.50, 70.15, 68.85, 66.26, 60.44, 70.79, 76.62, 68.21, 71.44, 66.26, 68.21, 72.74, 76.62, 60.44, 74.68, 63.03, 60.44, 72.09, 72.74, 74.68, 66.26, 71.44, 74.68, 75.97, 75.97, 53.97
1H NMR (400 MHz, D2O) δ 5.19, 5.09, 4.94, 4.92, 4.91, 4.83, 4.70, 4.64, 4.51, 4.44, 4.27, 4.06, 4.00, 3.92, 3.90, 3.85, 3.82, 3.81, 3.75, 3.73, 3.72, 3.71, 3.71, 3.71, 3.67, 3.62, 3.61, 3.60, 3.58, 3.53, 3.48, 3.44, 3.41, 3.39, 3.36, 3.24, 3.15.
f2 (ppm)
f1 (ppm)

(d)

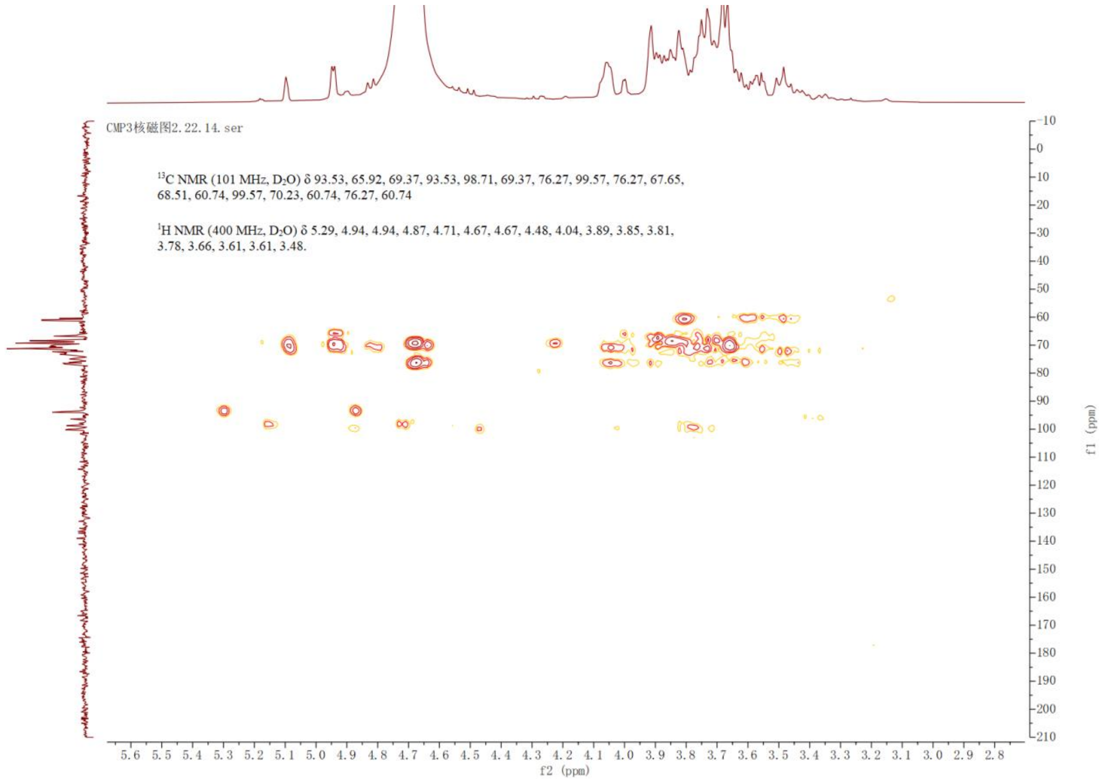

(e)

Figure S3. NMR analysis of FCMP 3: (a) ^{13}C NMR (b) ^{1}H NMR (c) COSY (d) HSQC (e) HMBC.

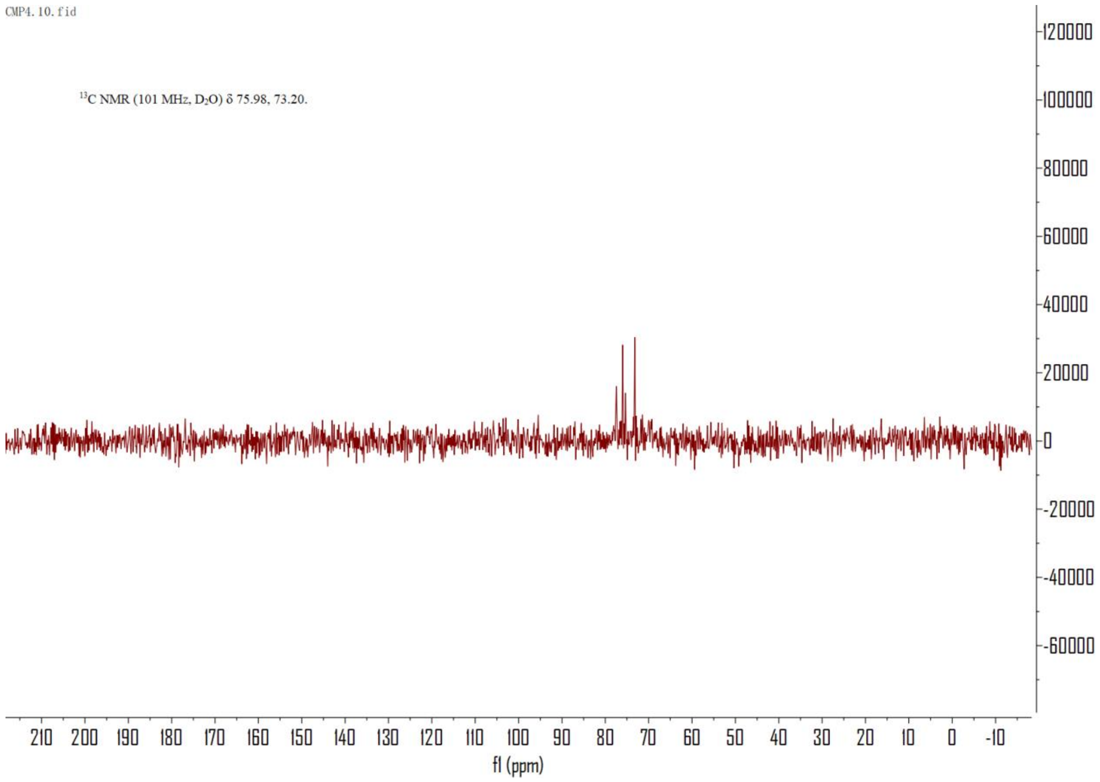
CMP4.10.fid
^{13}C NMR (101 MHz, D_2O) δ 75.98, 73.20.
120000
100000
80000
60000
40000
20000
0
-20000
-40000
-60000
210 200 190 180 170 160 150 140 130 120 110 100 90 80 70 60 50 40 30 20 10 0 -10
f1 (ppm)

(a)

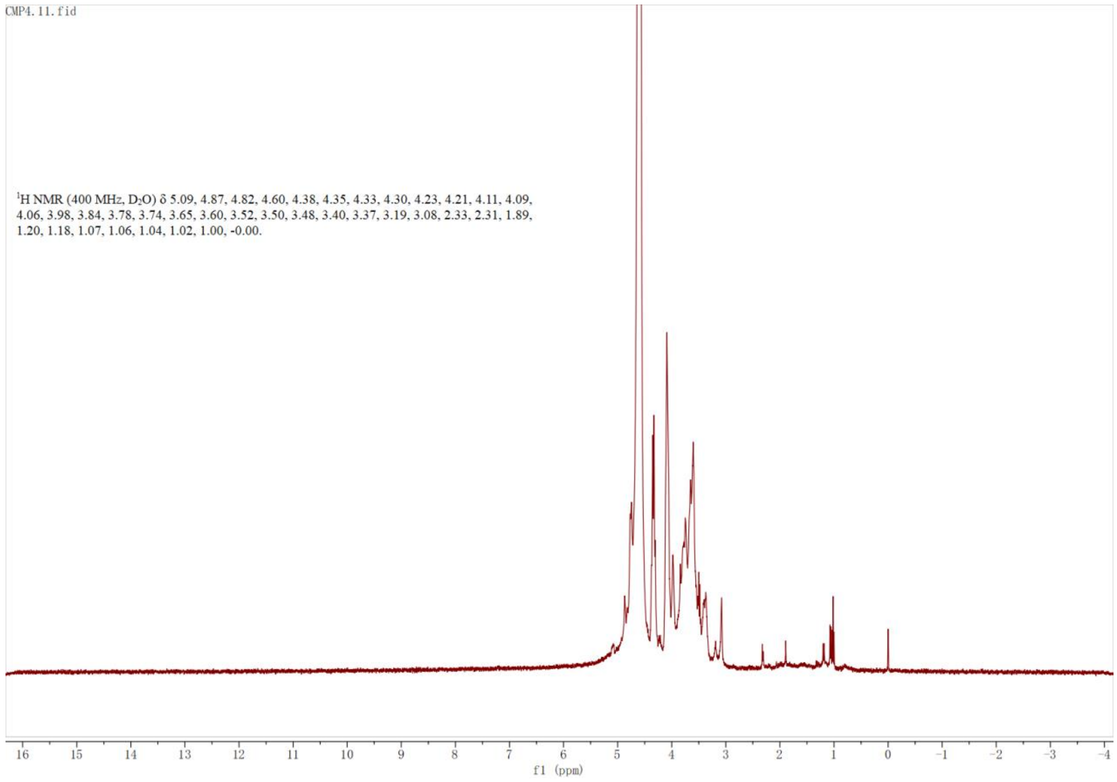
CMP4.11.fid
^{1}H NMR (400 MHz, D_2O) δ 5.09, 4.87, 4.82, 4.60, 4.38, 4.35, 4.33, 4.30, 4.23, 4.21, 4.11, 4.09, 4.06, 3.98, 3.84, 3.78, 3.74, 3.65, 3.60, 3.52, 3.50, 3.48, 3.40, 3.37, 3.19, 3.08, 2.33, 2.31, 1.89, 1.20, 1.18, 1.07, 1.06, 1.04, 1.02, 1.00, -0.00.
16 15 14 13 12 11 10 9 8 7 6 5 4 3 2 1 0 -1 -2 -3 -4
f1 (ppm)

(b)

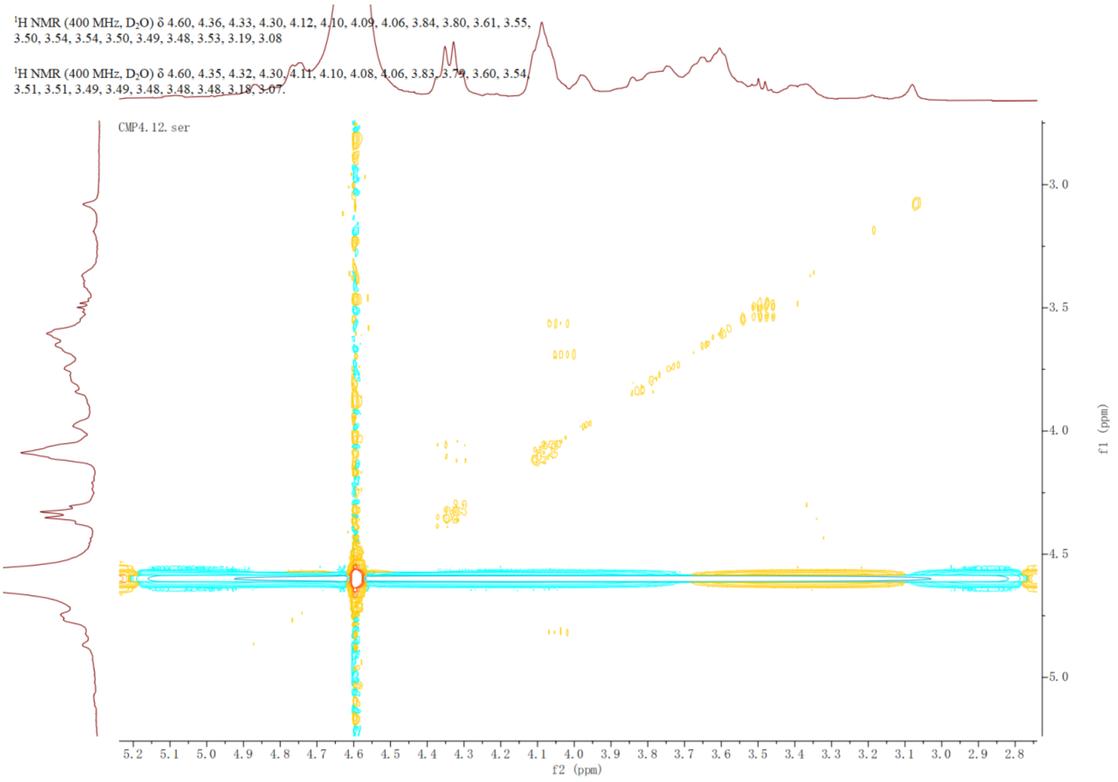

(c)

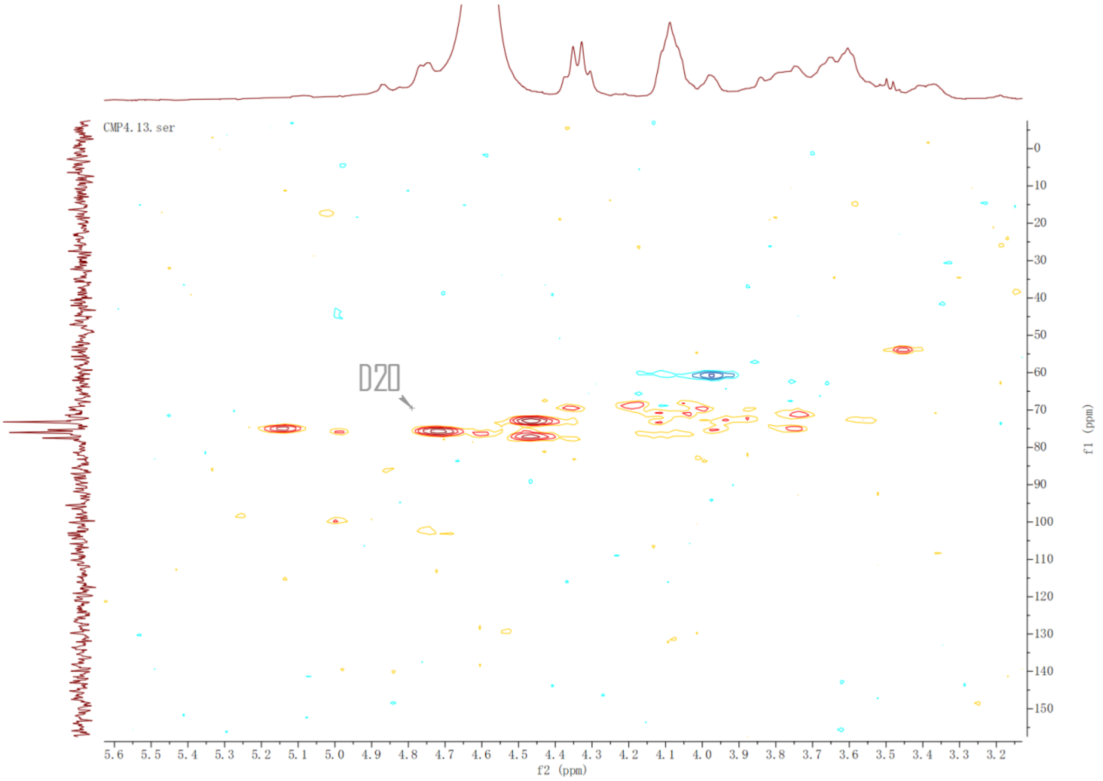

(d)

Figure S4. NMR analysis of FCMP 4: (a) ^{13}C NMR (b) ^{1}H NMR (c) COSY (d) HSQC

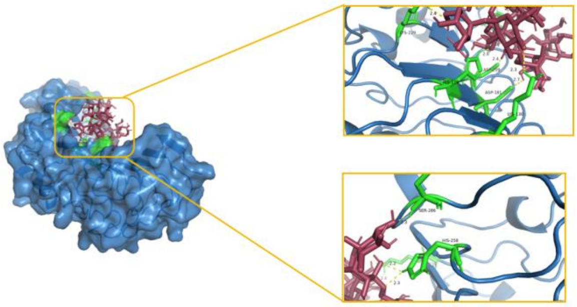

Figure SS1A. Molecular docking diagram of FCMP1 with the TLR4/MD-2 complex.

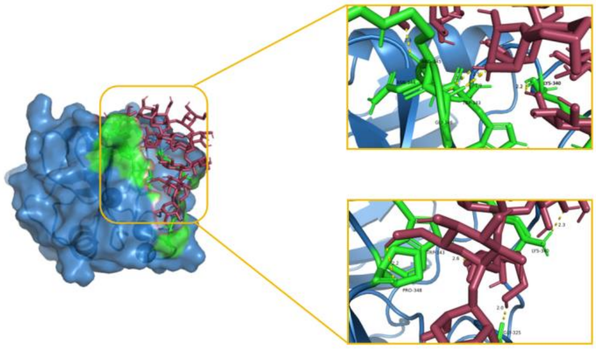

Figure SS1B. Molecular docking illustration of FCMP1 with DC-SIGN receptor.

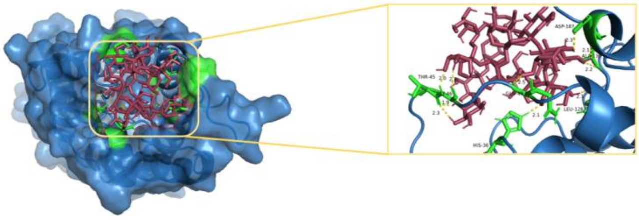

Figure SS1C. Molecular docking representation of FCMP1 with AQP1.

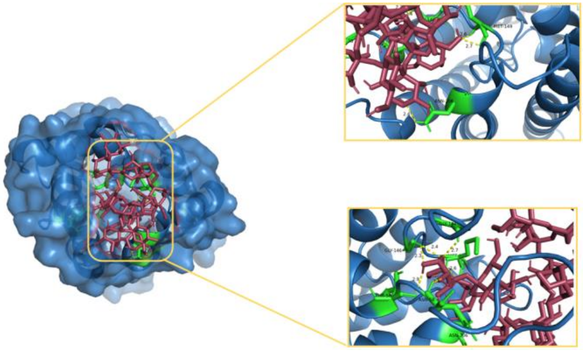

Figure SS1D. Molecular docking diagram of FCMP1 with AQP4.

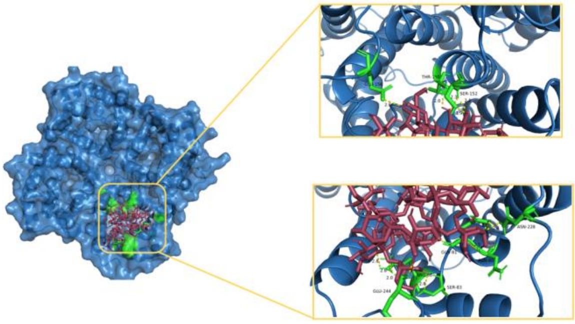

Figure SS1E. Molecular docking diagram of FCMP1 with AQP5.

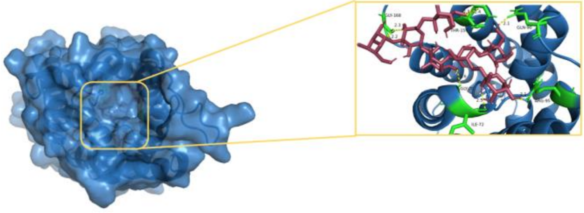

Figure SS2A. Molecular docking diagram of FCMP2 with AQP1.

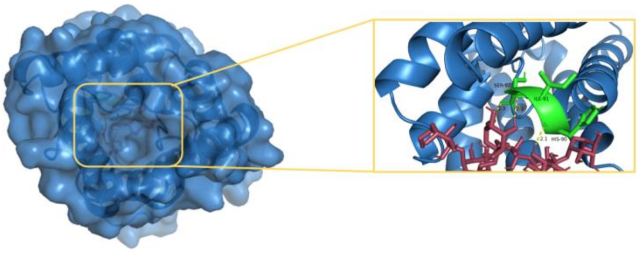

Figure SS2B. Molecular docking diagram of FCMP2 with AQP4.

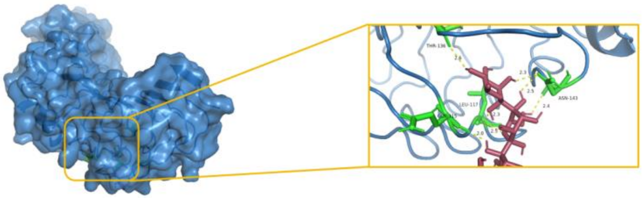

Figure SS3A. Molecular docking diagram of FCMP3 with TLR4.

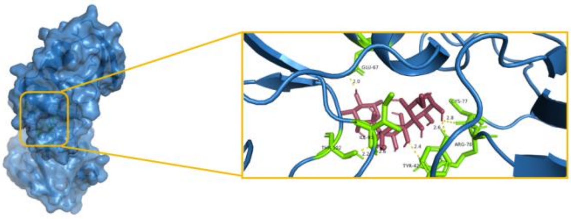

Figure SS3B. Molecular docking diagram of FCMP3 with CD44.

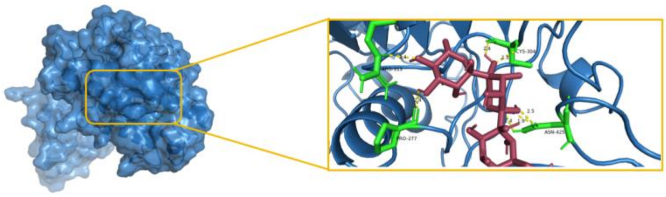

Figure SS3C. Molecular docking diagram of FCMP3 with pancreatic lipase.

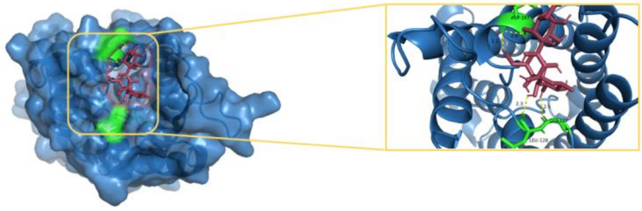

Figure SS3D. Molecular docking diagram of FCMP3 with AQP1.

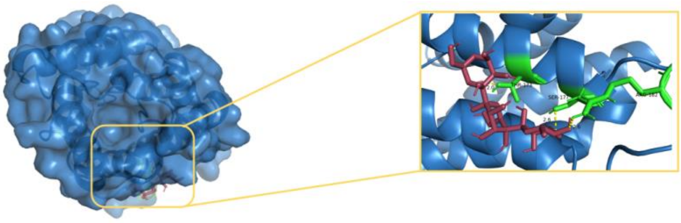

Figure SS3E. Molecular docking diagram of FCMP3 with AQP4.

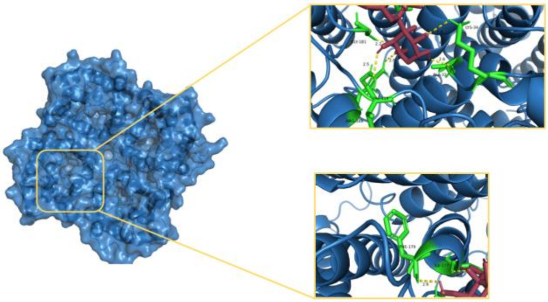

Figure SS3F. Molecular docking diagram of FCMP3 with AQP5.

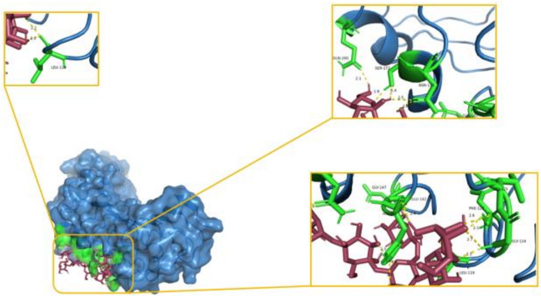

Figure SS4A. Molecular docking diagram of FCMP4 with TLR4.

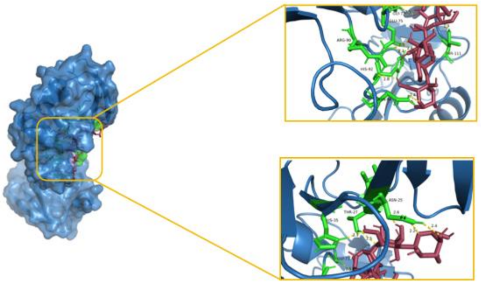

Figure SS4B. Molecular docking diagram of FCMP4 with CD44.

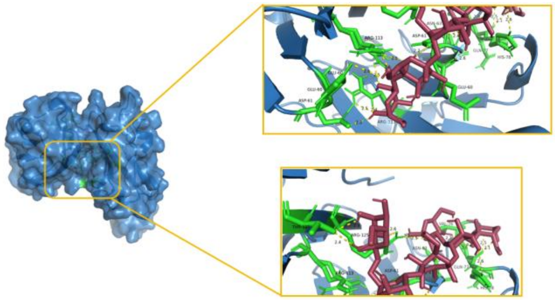

Figure SS4C. Molecular docking diagram of FCMP4 with PD-L1.

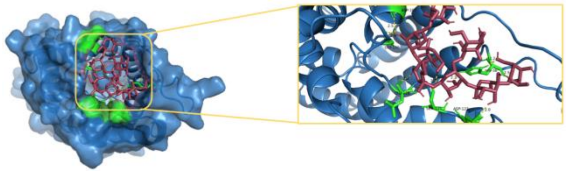

Figure SS4D. Molecular docking diagram of FCMP4 with AQP1.

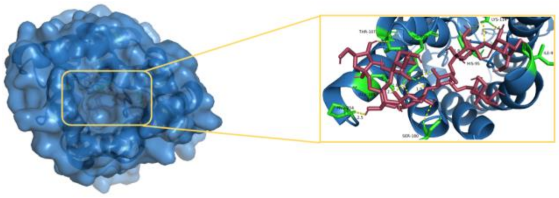

Figure SS4E. Molecular docking diagram of FCMP4 with AQP4.

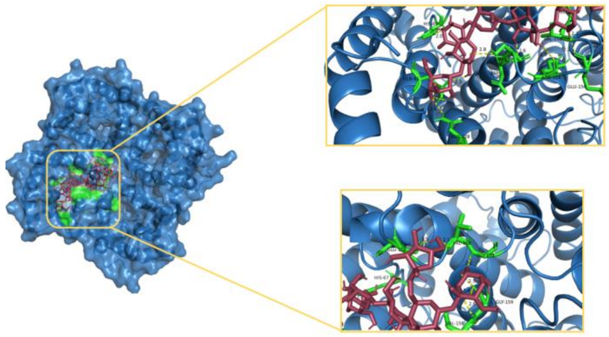

Figure SS4F. Molecular docking diagram of FCMP4 with AQP5.
